# Epilepsy enhance global efficiency of language networks in right temporal lobe gliomas

**DOI:** 10.1111/cns.13595

**Published:** 2021-01-19

**Authors:** Shengyu Fang, Yinyan Wang, Tao Jiang

**Affiliations:** ^1^ Beijing Neurosurgical Institute, Capital Medical University Beijing China; ^2^ Department of Neurosurgery Beijing Tiantan Hospital Capital Medical University Beijing China; ^3^ Research Unit of Accurate Diagnosis, Treatment, and Translational Medicine of Brain Tumors Chinese Academy of Medical Sciences Beijing China

**Keywords:** epilepsy, glioma, magnetic resonance imaging, neural networks

## Abstract

**Aims:**

We analyzed the resting state functional magnetic resonance images to investigate the alterations of neural networks in patients with glioma‐related epilepsy (GRE).

**Methods:**

Fifty‐six patients with right temporal lower‐grade glioma were divided into GRE (*n* = 28) and non‐GRE groups. Twenty‐eight healthy subjects were recruited after matching age, sex, and education level. Sensorimotor, visual, language, and left executive control networks were applied to generate functional connectivity matrices, and their topological properties were investigated.

**Results:**

No significant alterations in functional connectivity were found. The least significant discovery test revealed differences only in the language network. The shortest path length, clustering coefficient, local efficiency, and vulnerability were greater in the non‐GRE group than in the other groups. The nodal efficiencies of two nodes (mirror areas to Broca and Wernicke) were weaker in the non‐GRE group than in the other groups. The node of degree centrality (Broca), nodal local efficiency (Wernicke), and nodal clustering coefficient (temporal polar) were greater in the non‐GRE group than in the healthy group.

**Conclusion:**

Different tumor locations alter different neural networks. Temporal lobe gliomas in the right hemisphere altered the language network. Glioma itself and GRE altered the network in opposing ways in patients with right temporal glioma.

## Graphical abstract

Right temporal glioma will make the nodal efficiency of the left part of the language network decline and increased degree centrality, nodal local efficiency, and nodal clustering coefficient of left part of the language network.

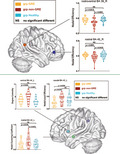

## 1 INTRODUCTION

Glioma‐related epilepsy (GRE) is a common symptom in patients with diffuse lower‐grade glioma (DLGG, World Health Organization grades 2 and 3),^1^, ^2^ especially in cases involving gliomas growing in the frontal or temporal lobes.^3^ Primary seizures are thought as a network‐related disorder. The correlations between alterations in functional networks and epilepsy have been reported.^4^, ^5^ However, alterations in functional networks in patients with GRE remain poorly understood.

The resting state functional magnetic resonance imaging (rs‐fMRI) acquires oxyhemoglobin signals when patients are in a resting state. The synchronization of the oxyhemoglobin signals between the two brain regions is used to delineate functional connectivity. Graph theoretical analysis is a quantitative measurement that reflects connective model of functional network and the ability to convey information.^6^, ^7^ In previous studies, the disruption of functional connectivity (FC) and weakening of network efficiency were induced via primary temporal lobe seizures, as primary seizures reduce the synchronous fluctuations between different cortices.^6^, ^8^, ^9^ Nevertheless, changes in neural networks were caused by both the glioma and GRE. Hence, previous conclusions based on primary seizures are not applicable in patients with glioma and may occlude appropriate preoperative prevention and intraoperative treatment. Although left temporal gliomas were found to activate visual networks and GRE to inhibit visual networks,^10^ it remains unknown whether right temporal gliomas and GRE cause the same changes. Consequently, further investigation of how the glioma in right temporal lobe and GRE impact neural networks is important to optimize strategies of preoperative seizure control and intraoperative treatment.

To bridge this knowledge gap, the patients with right temporal DLGG and healthy subjects were retrospectively recruited to investigate how the neural networks were altered by the right temporal glioma and GRE.

## 2 METHODS

This study was approved by the institutional review board of Beijing Tiantan Hospital and performed in accordance with the ethical standards put forth in the Declaration of Helsinki. All participants provided informed written consent before data acquisition.

### 2.1 Participants

The records of patients aged >18 years who underwent surgical treatment at the glioma treatment center of Beijing Tiantan Hospital between July 2017 and September 2020 were reviewed. The inclusion criteria were as follows: a) no history of brain disease, b) majority of glioma located on the right temporal hemisphere, c) histopathological diagnosis of primary DLGG according to the histopathological criteria (2016, World Health Organization,^11^), and d) only taking levetiracetam 0.5 g twice a day to control GRE once diagnosed as glioma. The exclusion criteria were as follows: a) head motion greater than 1 mm in translation or 1° in rotation, b) glioma invading the bilateral hemisphere, c) a period of levetiracetam administration exceeding 30 days, and d) contraindications for MRI.

### 2.2 Clinical characteristics

The information on age, sex, education time, Karnofsky performance score, extent of tumor resection, histopathology, isocitrate dehydrogenase mutation and chromosome 1p/19q codeletion, history of preoperative seizures, and performances during seizure onset was acquired from inpatient records. Follow‐up information was obtained by telephone interviews at 6 months after tumor resections.

### 2.3 MRI acquisition

We used a 3‐T MR scanner (MAGNETOM Prisma, Siemens, Erlangen, Germany) to acquire MR data. The T2 sequence with fluid‐attenuated inversion recovery (FLAIR) (echo time [TE], 87 ms; repetition time [TR], 3200 ms; field of view [FOV], 220 mm * 220 mm; fractional anisotropy [FA], 150°; voxel size, 0.9 mm * 0.9 mm * 5 mm; slice number, 25). Moreover, we used rs‐fMRI sequence to acquire functional image data (TE, 30 ms; TR, 2000 ms; FOV, 220 mm * 220 mm; FA, 75°; voxel size, 3.0 mm * 3.0 mm * 5.0 mm; slice number, 30; and acquisition time, 8 min.

All patients were scanned within 3 days before surgery.

### 2.4 Pipeline of rs‐MRI preprocessing

A software, Graph Theoretical Network Analysis (https://www.nitrc.org/projects/gretna), ^12^, ^13^ was used to process rs‐fMRI data. The pipeline was the same as in the previous study^10^ and is shown in the Appendix S1.

### 2.5 Regions of tumor invasion

Each glioma was segmented in its individual space based on the region with hyper‐intensity in T2‐FLAIR images. The extent of glioma infiltration was manually and independently determined by two neuroradiologists. If the determined regions varied more than 5%, the final decision was made by a third neuroradiologist who had over 20‐year clinical experience. Subsequently, all tumor masks were normalized into the standard space of the Montreal Neurological Institute template by using SPM8 software (http://www.fil.ion.ucl.ac.uk/spm/software/spm8).

### 2.6 Regions of interest

Regions of interest (ROIs) were generated from “brainnetome atlas” (http://www.brainnetome.org/).^14^ This open‐access atlas comprises 246 brain regions, to acquire matrices of FC. Four sub‐templates were extracted, which were sensorimotor, language, left executive control, and visual networks. The ROIs in these networks invaded by glioma were excluded. Hence, the inaccurate effect of registration was reduced as much as possible. Detailed information on each ROI in presented in Tables S1–S4.

### 2.7 Network construction

The mean time series between each two ROIs were compared using Pearson correlation, and subsequently, the FC matrices were constructed. Consequently, we obtained four different FC matrices.

### 2.8 Graph theoretical measurement

Topological properties of the four sub‐networks were analyzed using graph theory measurement, which included global properties (the shortest path length, global efficiency, local efficiency, clustering coefficient, transitivity, and vulnerability), nodal properties (nodal efficiency, nodal local efficiency, nodal clustering coefficient, degree centrality), and small‐worldness.^10^, ^15^, ^16^ The details of properties were shown in part 2 of the Appendix S1. All matrices were absolutized and binarized to further analyze the topological properties.

### 2.9 Statistical analyses

Epidemiology characteristics were compared among the GRE, non‐GRE, and healthy groups by using Student's *t* test, Mann‐Whitney *U* test, chi‐squared test, Fisher's exact test, and one‐way analysis of variance (one‐way ANOVA) based on categories of data. All data were tested to ensure whether they were normal/Gaussian distribution. If a group of data did not exhibit a normal distribution, a Student *t* test or one‐way ANOVA test was applied with a non‐parametric equivalent.

The differences in FC of the four sub‐networks were generated from comparisons between the patient and healthy groups using Student's *t* test. Moreover, false discovery rate (FDR) was applied to correct the generated results. To found differences in topological properties, we used a series of sparsity thresholds (from 0.17 to 0.33, interval 0.01) consistent with a previously study.^4^ For each subject, topological properties were generated according to sparsity. Each property was first analyzed using one‐way ANOVA test. Subsequently, post hoc pairwise comparisons were performed on the generated results in global and nodal properties with least significant difference (LSD) test. A significant *p*‐value was lower than 0.05.

## 3 RESULTS

### 3.1 Demographic characteristics

Fifty‐six patients met the inclusion criteria, and four patients were excluded, as their periods of anti‐epileptic drug use were longer than 30 days. According to the history of preoperative GRE onset, 28 patients were divided into the GRE group (male, *n* = 11) and the others into the non‐GRE group (male, *n* = 14, Table 1). All patients were right‐handed according to the assessments by the Edinburgh Handedness Inventory test, and their epilepsy onset performance was considered as a secondary generalized epilepsy. Our postoperative follow‐up showed that all patients achieved Engel class I at 6 months after tumor resection. Based on the sample size of each group, 28 healthy participants were finally recruited after matching age, sex, and education level (14 males; all right‐handed).

**TABLE 1:** Demographic and clinical characteristics of patient groups

Demographic and clinical characteristics	GRE (*n* = 28)	Non‐GRE (*n* = 28)	Healthy (*n* = 28)	*p*‐value
Gender				
Male	11	14	14	0.65
Female	17	14	14	
Age (y)*	41.4 ± 2.3	46.0 ± 2.2	39.7 ± 1.7	0.09
Handedness				
Right	28	28	28	‐
Left	0	0	0	
KPS score (preoperative)				
100	24	26	28	0.12
90 ~ 100	4	2	0	
Education level (y)*	13.0 ± 0.6	12.0 ± 0.6	13.4 ± 0.6	0.16
Histopathology				
Astrocytoma	8	6	‐	0.73
Oligodendroglioma	4	7	‐	
Anaplastic astrocytoma	13	13	‐	
Anaplastic oligodendroglioma	3	2	‐	
IDH status				
Mutation	11	13		0.59
Wild‐type	17	15		
Chromosome 1p/19q status				
Codeletion	6	8		0.53
Non‐codeletion	22	20		
Tumor volume (ml)*	52.38 ± 7.06	49.65 ± 4.98	‐	0.85
Onset age (y)*	41.1 ± 2.1	‐	‐	‐
Frequency before diagnosis				
Low (only once)	23			
Medium (2 ~ 3 times)	3			
High (>3 times)	2			
Preoperative anti‐epileptic drugs				
Levetiracetam (0.5 g, twice a day)	28			‐
Postoperative epileptic control				
Engel class I	28			‐

Note

Using Mann‐Whitney *U* test to compare the difference of Karnofsky performance status between GRE and non‐GRE groups.

Using Student *t* test via non‐parametric equivalent to compare the difference of tumor volume between GRE and non‐GRE groups.

Using one‐way ANOVA test to compare the difference of age between patients groups and healthy group.

Using one‐way ANOVA test via non‐parametric equivalent to compare the difference of education level between patients groups and healthy group.

Using to chi‐square test to compare the differences of gender, tumor location, and IDH status between GRE and non‐GRE groups.

Abbreviation: KPS, Karnofsky performance status.

* Values are means ±standard error of mean.

No differences in Karnofsky performance score (*p* = 0.12, Mann‐Whitney *U* test), ratio of histopathology (*p* = 0.73), isocitrate dehydrogenase mutation (*p* = 0.59, chi‐squared test), or chromosome 1p/19q codeletion (*p* = 0.53, chi‐squared test) were observed between the GRE and non‐GRE groups. Moreover, no difference in tumor volume (*p* = 0.75) was found between the GRE (52.38 ± 7.06 ml) and non‐GRE groups (49.65 ± 4.98 ml).

### 3.2 Functional connectivity differences

Our results revealed no differences in FC of the four sub‐networks (sensorimotor, visual, language, and left executive control networks) among the three groups after FDR correction.

### 3.3 Differences in global topological properties

In the language network, the clustering coefficient (*p* = 0.0070), global efficiency (*p* < 0.0001), local efficiency (*p* = 0.0045), shortest path length (*p* < 0.0001), transitivity (*p* = 0.0002), and vulnerability (*p* = 0.0499) were different among the three groups, as determined using one‐way ANOVA (Table S5).

**FIGURE 1:**
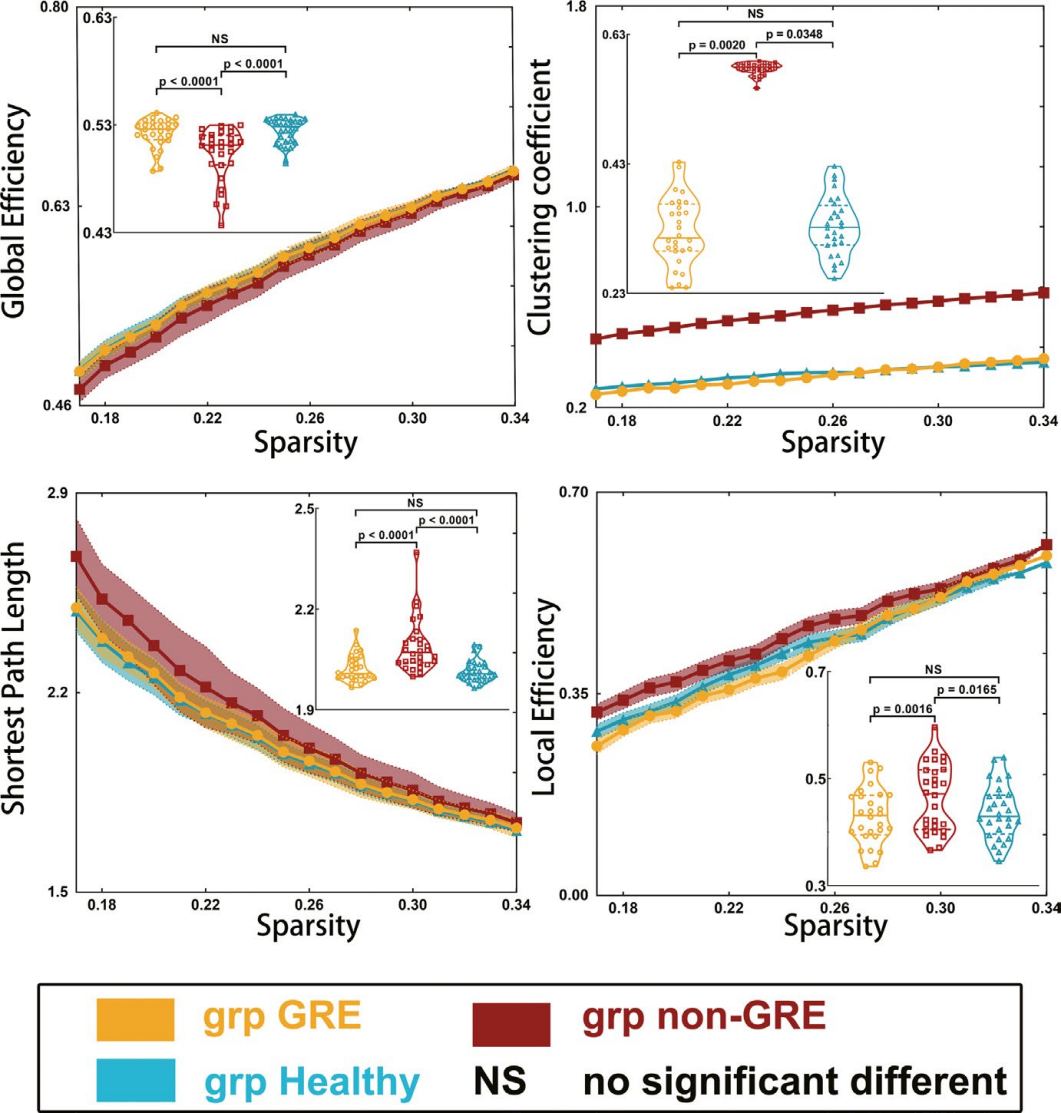
Results of alterations in global topological properties when gliomas grew in the right temporal lobe. The grp GRE = group of patients with glioma‐related epilepsy. The grp non‐GRE = group of patients without glioma‐related epilepsy. The grp healthy = group of healthy participants

Post hoc analysis with the LSD test (Figure 1) revealed that the non‐GRE group exhibited weaker global efficiency (0.502 ± 0.005) than the GRE (0.522 ± 0.003, *p* < 0.0001) and control groups (0.525 ± 0.002, *p* < 0.0001). Moreover, the non‐GRE group showed greater local efficiency (0.465 ± 0.012) than the GRE (0.430 ± 0.010, *p* = 0.0016) and control groups (0.437 ± 0.010, *p* = 0.0165). The non‐GRE group exhibited a longer shortest path length (2.089 ± 0.015) than the GRE (2.023 ± 0.008, *p* < 0.0001) and control groups (2.015 ± 0.006, *p* < 0.0001). Furthermore, the non‐GRE group showed a greater clustering coefficient (0.577 ± 0.002) than the GRE (0.325 ± 0.010, *p* = 0.0020) and control groups (0.334 ± 0.009, *p* = 0.0348). No differences in global efficiency (*p* = 0.5939), local efficiency (*p* = 0.4157), shortest path length (*p* = 0.6025), or clustering coefficient (*p* = 0.9079) were found between the GRE and control groups.

**FIGURE 2:**
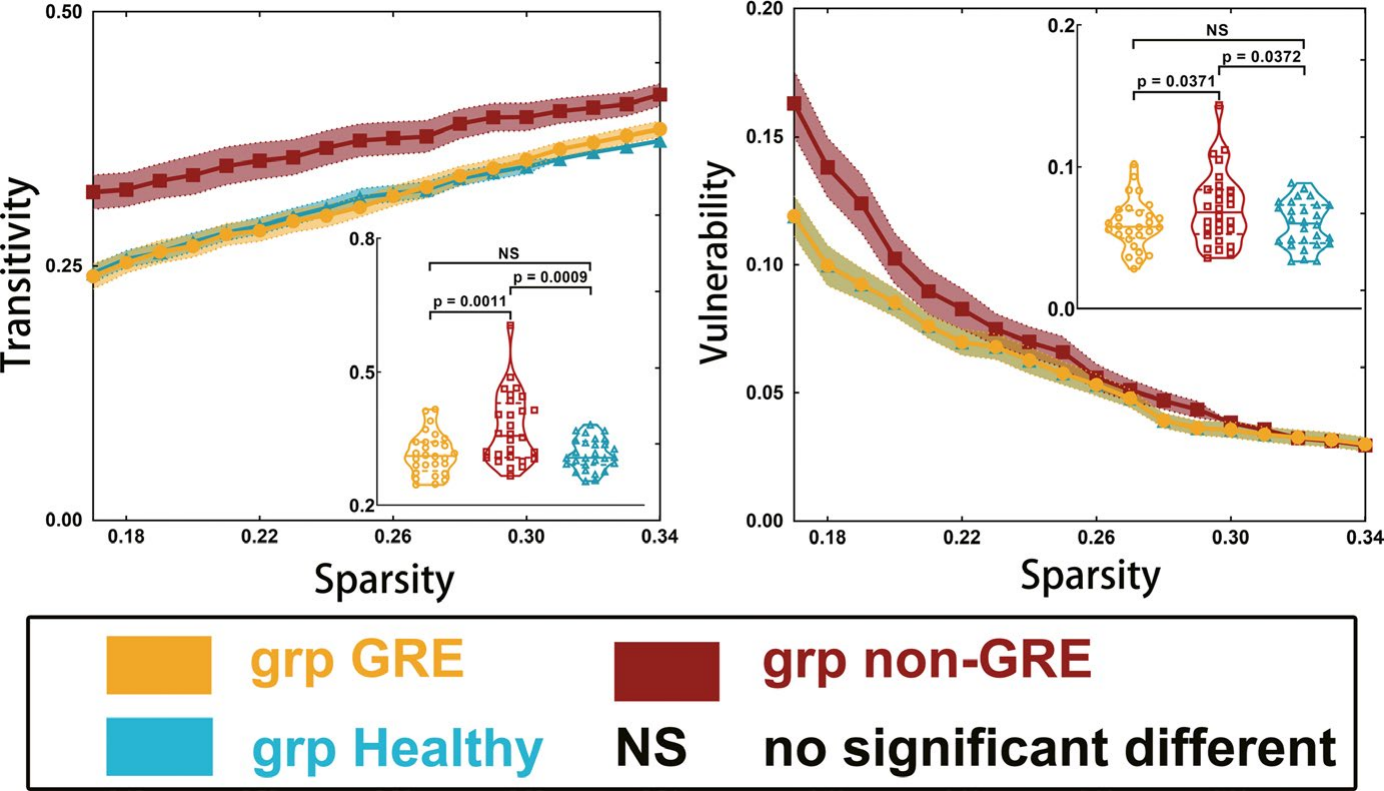
Results of alterations in transitivity and vulnerability when gliomas grew in the right temporal lobe. The grp GRE = group of patients with glioma‐related epilepsy. The grp non‐GRE = group of patients without glioma‐related epilepsy. The grp healthy = group of healthy participants

Post hoc analysis using the LSD test (Figure 2) revealed that the non‐GRE group had greater transitivity (0.372 ± 0.015) than the GRE (0.316 ± 0.009, *p* = 0.0011) and control groups (0.315 ± 0.007, *p* = 0.0009). Moreover, the non‐GRE group had more severe vulnerability (0.071 ± 0.005) than the GRE (0.060 ± 0.003, *p* = 0.0371) and control groups (0.060 ± 0.003, *p* = 0.0371). No differences in transitivity (*p* = 0.9524) or vulnerability (*p* = 0.9999) were found between the GRE and control groups.

No differences in global topological properties were found in the other three sub‐networks (sensorimotor, visual, and left executive networks).

### 3.4 Differences in small‐worldness properties

**FIGURE 3:**
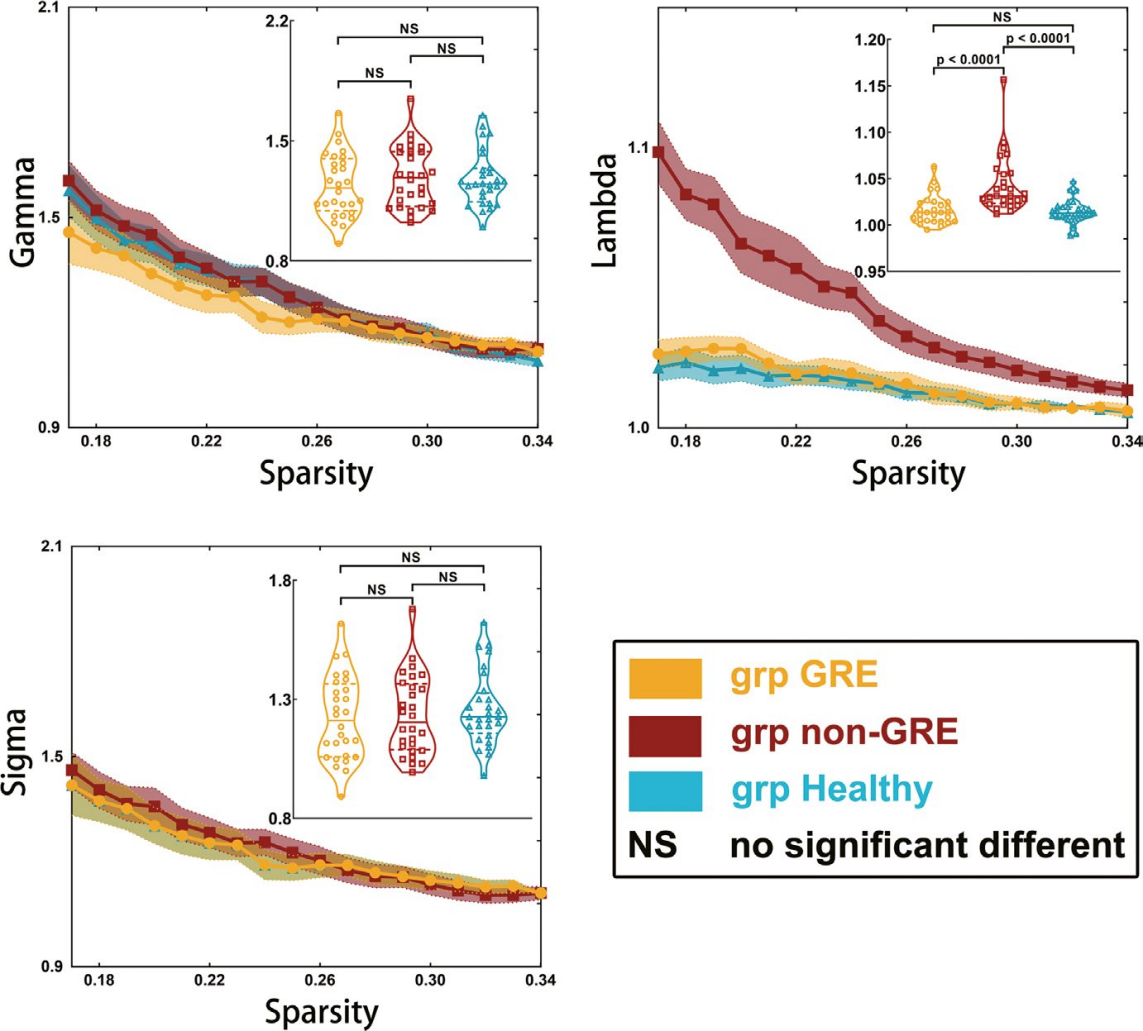
Results of alterations in small‐worldness when gliomas grew in the right temporal lobe. The grp GRE = group of patients with glioma‐related epilepsy. The grp non‐GRE = group of patients without glioma‐related epilepsy. The grp healthy = group of healthy participants

In the language network, the value of lambda (*p* < 0.0001) differed among the groups, as determined using one‐way ANOVA (Table S5 and Figure 3). No differences in the values of gamma (*p* = 0.4822) or sigma (*p* = 0.5176) were found among the three groups. Post hoc analysis using the LSD test showed that the non‐GRE group exhibited a higher value of lambda (1.043 ± 0.006) than the GRE (1.016 ± 0.003, *p* < 0.0001) and control groups (1.014 ± 0.002, *p* < 0.0001). No difference in the value of lambda (*p* = 0.5969) was found between the GRE and control groups.

No significant alterations in small‐worldness (gamma, lambda, and sigma) in the other three sub‐networks (sensorimotor, visual, and left executive networks) were found among the three groups.

### 3.5 Differences in nodal topological properties

**FIGURE 4:**
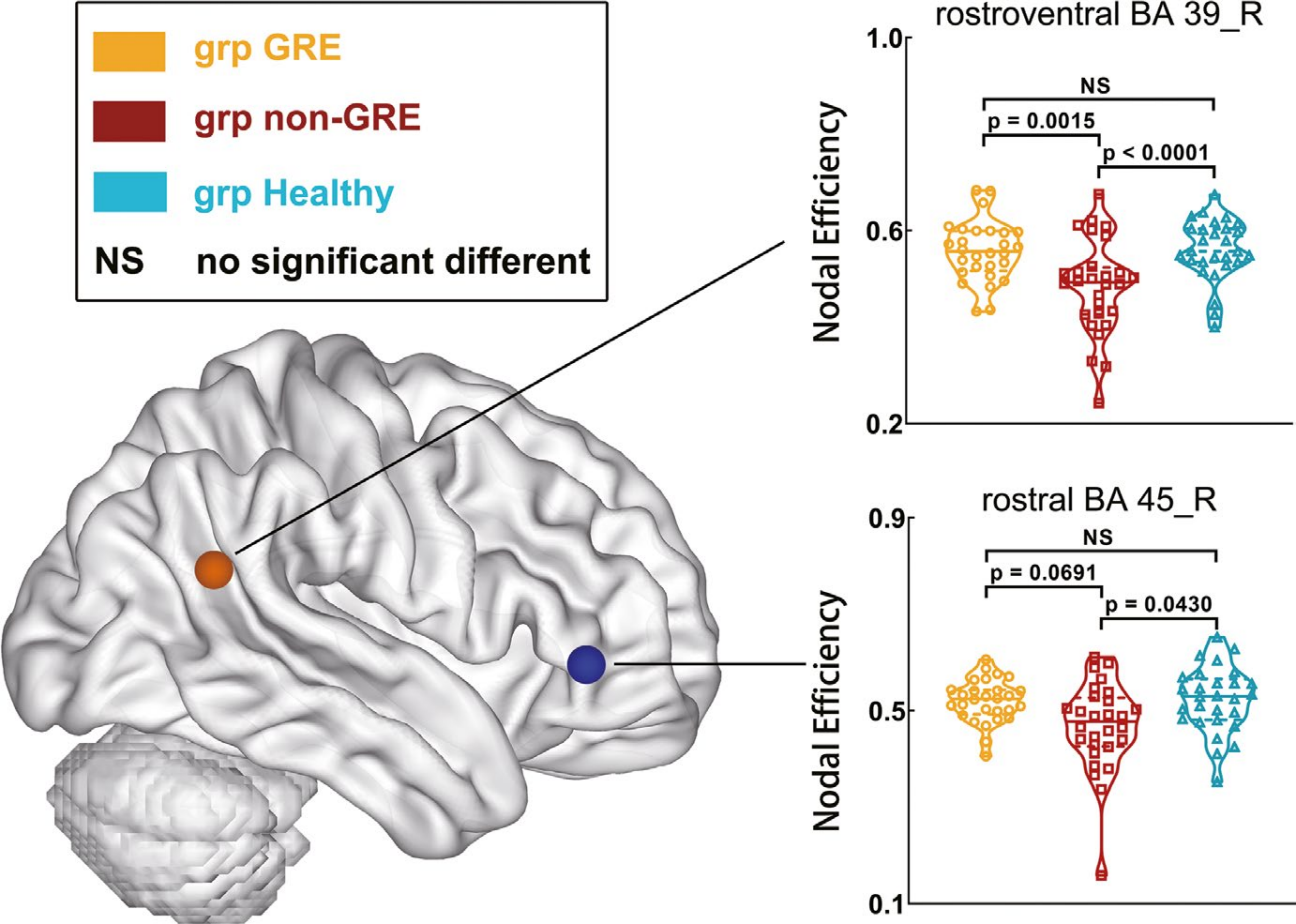
Results of alterations in nodal properties of right nodes in the language network when gliomas grew in the right temporal lobe. The grp GRE = group of patients with glioma‐related epilepsy. The grp non‐GRE = group of patients without glioma‐related epilepsy. The grp healthy = group of healthy participants

One‐way ANOVA revealed two nodes in the right hemisphere that had differing nodal efficiencies among the three groups in the language network (Table S6 and Figure 4): rostroventral BA 39 (A39rv_R, *p* = 0.0002) and rostral BA 45 (A45r_R, *p* = 0.0060). Regarding A39rv_R, the non‐GRE group had weaker nodal efficiency (0.483 ± 0.019) than the GRE (0.558 ± 0.012, *p* = 0.0014) and control groups (0.560 ± 0.012, *p* = 0.0010) after post hoc analysis. Similarly, regarding A45r_R, the non‐GRE group showed weaker nodal efficiency (0.467 ± 0.017) than the GRE (0.520 ± 0.008, *p* = 0.0207) and control groups (0.523 ± 0.013, *p* = 0.0129) after post hoc analysis. No differences in nodal efficiency of these two nodes were found between the GRE and control groups (A39rv_R, *p* = 0.8903 and A45r_R, *p* = 0.8693).

**FIGURE 5:**
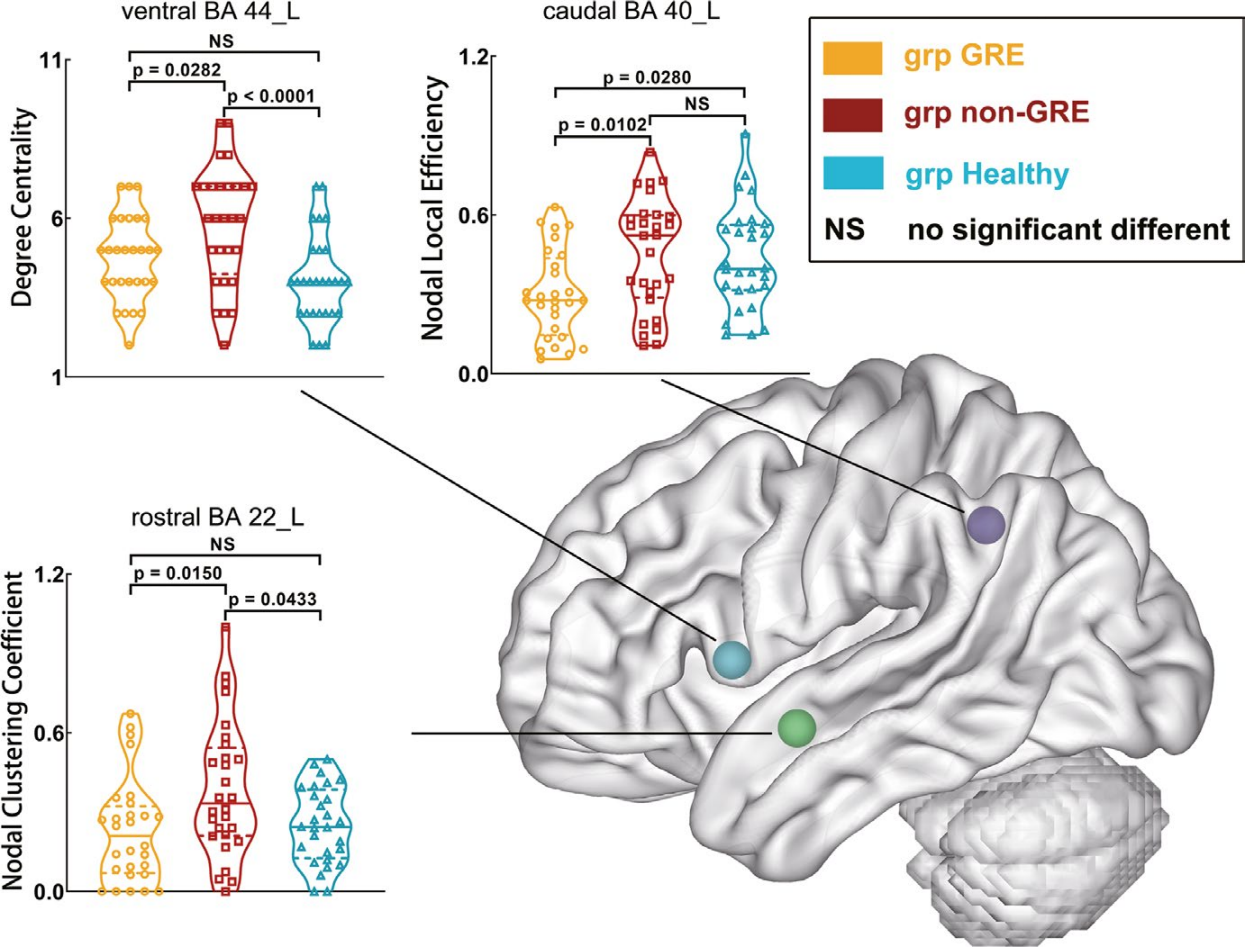
Results of alterations in nodal properties of left nodes in the language network when gliomas grew in the right temporal lobe. The grp GRE = group of patients with glioma‐related epilepsy. The grp non‐GRE = group of patients without glioma‐related epilepsy. The grp healthy = group of healthy participants

Among the three groups, some differences in nodal local efficiency, degree centrality, and nodal clustering coefficient were found in caudal BA 40 (A40c_L, *p* = 0.0061), ventral BA 44 (A44v_L, *p* = 0.0007), and rostral BA 22 (A22r_L, *p* = 0.0097) in the left hemisphere, as determined using one‐way ANOVA (Tables S7–S9 and Figure 5). Regarding A40c_L, the GRE group exhibited weaker nodal local efficiency (0.301 ± 0.032) than the non‐GRE (0.455 ± 0.039, *p* = 0.0102) and control groups (0.437 ± 0.037, *p* = 0.0280) after post hoc analysis. Regarding A44v_L, the non‐GRE group exhibited greater degree centrality (5.821 ± 0.349) than the GRE (4.750 ± 0.250, *p* = 0.0282) and control groups (4.071 ± 0.262, *p* = 0.0005) after post hoc analysis. With regard to A22r_L, the non‐GRE group exhibited a greater nodal clustering coefficient (0.386 ± 0.048) than the GRE (0.229 ± 0.037, *p* = 0.0150) and control groups (0.250 ± 0.027, *p* = 0.0433) after post hoc analysis.

No significant alterations in nodal topological properties in the other three sub‐networks (sensorimotor, visual, and left executive networks) were found among the three groups.

## 4 DISCUSSION

In this study, we investigated alterations in functional neural networks induced by right temporal GRE. Our findings indicated that GRE and right temporal DLGGs resulted in altered language networks. Although the altered network differed from the left temporal GRE change (visual network), the trend of right temporal DLGGs and GRE‐induced functional network change was the same as that of the left. That is, the GRE‐induced functional network change was found to be opposite of that induced by DLGG.

Global efficiency and shortest path length reflect the ability and cost of conveying information, respectively.^17^ In our findings, right temporal glioma decreased global efficiency and increased the shortest path length of the language network in the non‐GRE group. These changes were related to a neural pathway disruption caused by glioma infiltration. Indeed, the main language network is located in the left hemisphere in right‐handed people.^18^ The fMRI results suggested that when the left language network was damaged, functional compensation occurred in the cortex of the right hemisphere corresponding to the left language regions.^19^ These findings indicate that parts of the language network are located on the right and cooperated with the left network to accomplish language tasks.^20^, ^21^ Consequently, if the right‐sided language network was damaged by glioma, the global efficiency of the whole language network was decreased in the non‐GRE group. Indeed, compared with the GRE and healthy groups, the clustering coefficient and local efficiency in the non‐GRE group were increased. These alterations reflect the increase in the number of functional connections, but this does not mean that the pathway between two nodes was shortened or the ability to convey information was increased. Moreover, the right temporal glioma failed to disrupt the main language network (left hemisphere) and was unable to further induce language deficits. Hence, the language network did not drastically reorganize in the non‐GRE group. Therefore, we infer that the global decline in the efficiency of the language network was the result of the damage caused by glioma, which increased the burden of the residual language network.

However, in the GRE group, global efficiency did not differ from that in the healthy group. Why was there no decrease in global efficiency in the GRE group? These alterations were associated with GRE‐induced network reorganization, but they did not indicate that GRE facilitated recovery of the language network. Unlike primary epilepsy with a long and frequent onset history, the period from onset of GRE to glioma diagnosis and tumor resection is short. Hence, cortical sclerosis,^22^ gray‐matter atrophy,^23^ and cortical hypo‐metabolism^24^ did not occur in patients with GRE. Conversely, we found that the path length in the GRE group was shorter than that in the non‐GRE group. The GRE shortens pathways to decrease system response time, which facilitates the rapid spread of local epileptic discharges.^25^, ^26^ Thus, we concluded that the mechanism of GRE altering the language network was as follows: the right temporal glioma first disrupted the right‐sided language network and decreased global efficiency; then, accompanied by the GRE, the residual network was reorganized with increased global efficiency.

Vulnerability represents the degree of global efficiency alteration when a node is replaced and reflects whether a neural network is stable.^27^ We observed that the vulnerability in the non‐GRE group was higher than that in the GRE and control groups. This finding indicated that the right temporal glioma rendered the residual language network vulnerable, and to maintain the residual language function normally, none of the nodes could be further broken or replaced. Simultaneously, this finding verified that GRE facilitated network reorganization to regain stability.

Similar results were found for nodal properties. The decreasing nodal efficiency of the right inferior frontal and supramarginal gyri in the non‐GRE group showed that the glioma damaged the original right language network and affected the two important nodes needed to process language information. Simultaneously, the left nodes in the language network had to increase the degree centrality (Broca area), nodal local efficiency (Wernicke area), and nodal clustering coefficient (in the left temporal lobe) to maintain language functions. However, influenced by both glioma and GRE, the alterations in these nodal properties were alleviated in the GRE group.

Gliomas located in different hemispheres will affect different neural networks, but the GRE and glioma itself induced the same network alterations. Based on our findings, the right temporal glioma and GRE affected the language network rather than the visual network, which was shown to be affected by left temporal glioma and GRE in a previous study.^10^ We thought that the different affected networks were related to the dominant hemisphere. When glioma is located in the left hemisphere, the main language network is damaged and residual language network reorganization occurs, whether caused by the glioma itself or GRE. Hence, no differences in language network alterations were found between the GRE and non‐GRE groups in the previous study. Regarding the right temporal glioma, the main language network was not affected. Therefore, the alterations in the language network caused by the glioma itself and the GRE were significantly different. A common trend is that the changes in the neural networks caused by glioma itself or GRE are converse, regardless whether the tumor is located on the left or right hemisphere.

## 5 LIMITATIONS

The phenomenon that levetiracetam normalized FC was found in patients with primary epilepsy who took levetiracetam over 3 months.^28^ In our GRE group, all patients indeed took levetiracetam, but the period of administration was short (not longer than 15 days). To our knowledge, no study has revealed whether taking levetiracetam in a short period would alter topological properties. Hence, we could not determine whether alterations of topological properties in patients with GRE tended to normalize due to levetiracetam administration. In the future, we will enroll relevant patients to investigate whether taking levetiracetam for a short period can induce alterations of the functional network in patients with GRE and validate the findings in this study.

## 6 CONCLUSIONS

Different tumor locations alter different neural networks. Temporal lobe gliomas in the right hemisphere altered the language network. Alterations in the language network caused by GRE were opposite to those caused by glioma itself. Our findings provide a novel insight into the GRE impact and improve our understanding of alterations in functional neural networks in patients with glioma. In addition, under the premise of protecting the language function, postoperative epileptic onset might be effectively controlled by electrical cauterizing the pia mater in the language network in patients with GRE.

## CONFLICTS OF INTEREST

All authors do not have any conflict of interest.

## AUTHOR CONTRIBUTION

SF, YW, and TJ. conceptualized and designed the study. SF and YW. acquired and analyzed the data, involved in statistics/verified analytical method, and wrote the final draft. YW and TJ. supervised the study. All authors read and approved final version.

## Supporting information

Tab S1–S9

App S1

## Data Availability

Anonymized data will be made available on request.
