# Supplementary material for: Epilepsy enhance global efficiency of language networks in right temporal lobe gliomas
Source: CNS Neurosci Ther. 2021 Jan 19;27(3):363–71. doi: 10.1111/cns.13595 (PMC7871790; doi:10.1111/cns.13595)
Supplement: Supplementary file 1 — Tab S1–S9 [file CNS-27-363-s001.pdf]

CNS Neuroscience & Therapeutics

Epilepsy-related brain network alterations in patients with temporal lobe glioma in the right hemisphere

Journal:	*CNS Neuroscience & Therapeutics*
Manuscript ID	CNSNT-2020-557.R1
Wiley - Manuscript type:	Original Article
Date Submitted by the Author:	16-Dec-2020
Complete List of Authors:	Fang, Shengyu; Beijing Neurosurgical Institute<br>Wang, Yinyan; Beijing Neurosurgical Institution<br>Jiang, Tao; Beijing Neurosurgical Institute,
Keywords:	glioma, epilepsy, magnetic resonance imaging, neural networks
Scope of Manuscript:	brain tumor, Cerebral cortex, Epilepsy, Glioma

SCHOLARONE™
Manuscripts

Title: Epilepsy-related brain network alterations in patients with temporal lobe glioma in the right hemisphere

Running Title: Glioma-related epilepsy alters neural networks

Author list:

Shengyu Fang, MD[1, 2]; Yinyan Wang, MD[1,2,†]; Tao Jiang, MD, PhD[1,2,3,†]

[1] Beijing Neurosurgical Institute, Beijing, China;

[2] Department of Neurosurgery, Beijing Tiantan Hospital, Capital Medical University, Beijing, China;

[3] Research Unit of Accurate Diagnosis, Treatment, and Translational Medicine of Brain Tumors Chinese Academy of Medical Sciences

† Co-corresponding Authors:

1. Tao Jiang, MD, PhD

Department of Neurosurgery, Beijing Tiantan Hospital, Capital Medical University, 119, the Western Road of the Southern 4th Ring Road, Beijing, China.

Postal code: 100070

Tel/Fax: +86-01059976689.

E-mail: taojiang1964@163.com

2. Yinyan Wang, MD

Department of Neurosurgery, Beijing Tiantan Hospital, Capital Medical University, 119, the Western Road of the Southern 4th Ring Road, Beijing, China.

Postal code: 100070

Tel/Fax: +86-01059976686.

E-mail: tiantanyinyan@126.com

Author Contributions:

Study concept and design: SF, YW, and TJ.

Data acquisition and analysis: SF and YW.

Statistics/verified analytical method: SF and YW.

Writing the first draft: SF and YW.

Supervision study: YW and TJ.

Read and approved final version: All authors.

Abstract

Aims: We analyzed the resting-state functional magnetic resonance images to investigate the alterations of neural networks in patients with glioma-related epilepsy (GRE).

Methods: Fifty-six patients with right temporal lower-grade glioma were divided into GRE (n = 28) and non-GRE groups. Twenty-eight healthy subjects were recruited after matching age, sex, and education level. Sensorimotor, visual, language, and left executive control networks were applied to generate functional connectivity matrices, and their topological properties were investigated.

Results: No significant alterations in functional connectivity were found. The least significant discovery test revealed differences only in the language network. The shortest path length, clustering coefficient, local efficiency, and vulnerability were greater in the non-GRE group than in the other groups. The nodal efficiencies of two nodes (mirror areas to Broca and Wernicke) were weaker in the non-GRE group than in the other groups. The node of degree centrality (Broca), nodal local efficiency (Wernicke), and nodal clustering coefficient (temporal polar) were greater in the non-GRE group than in the healthy group.

Conclusion: Different tumor locations alter different neural networks. Temporal-lobe gliomas in the right hemisphere altered the language network. Glioma itself and GRE altered the network in opposing ways in patients with right-temporal glioma.

Keywords: glioma, epilepsy, magnetic resonance imaging, neural networks

Introduction

Glioma-related epilepsy (GRE) is a common symptom in patients with diffuse lower-grade glioma (DLGG, World Health Organization grades 2 and 3), [1, 2] especially in cases involving gliomas growing in the frontal or temporal lobes.[3] Primary seizures are thought as a network-related disorder. The correlations between alterations in functional networks and epilepsy have been reported.[4, 5] However, alterations in functional networks in patients with GRE remain poorly understood.

The resting-state functional magnetic resonance imaging (rs-fMRI) acquires oxyhemoglobin signals when patients are in a resting state. The synchronization of the oxyhemoglobin signals between the two brain regions is used to delineate functional connectivity. Graph theoretical analysis is a quantitative measurement that reflects connective model of functional network and the ability to convey information.[6, 7] In previous studies, the disruption of functional connectivity (FC) and weakening of network efficiency were induced via primary temporal-lobe seizures, as primary seizures reduce the synchronous fluctuations between different cortices.[6, 8, 9] Nevertheless, changes in neural networks were caused by both the glioma and GRE. Hence, previous conclusions based on primary seizures are not applicable in patients with glioma and may occlude appropriate preoperative prevention and intraoperative treatment. Although left-temporal gliomas were found to activate visual networks and GRE to inhibit visual networks,[10] it remains unknown whether right-temporal gliomas and GRE cause the same changes. Consequently, further investigation of how the glioma in right temporal lobe and GRE impact neural networks is important to optimize strategies of preoperative seizure control and intraoperative treatment.

To bridge this knowledge gap, the patients with right temporal DLGG and healthy subjects were retrospectively recruited to investigate how the neural networks were

altered by the right-temporal glioma and GRE.

Methods

This study was approved by the institutional review board of Beijing Tiantan Hospital and performed in accordance with the ethical standards put forth in the Declaration of Helsinki. All participants provided informed written consent before data acquisition.

Participants

The records of patients aged > 18 years who underwent surgical treatment at the glioma treatment center of Beijing Tiantan Hospital between July 2017 and September 2020 were reviewed. The inclusion criteria were as follows: a) no history of brain disease, b) majority of glioma located on the right temporal hemisphere, c) histopathological diagnosis of primary DLGG according to the histopathological criteria (2016, World Health Organization, [11]) and d) only taking levetiracetam 0.5 g twice a day to control GRE once diagnosed as glioma. The exclusion criteria were as follows: a) head motion greater than 1 mm in translation or 1° in rotation, b) glioma invading the bilateral hemisphere, c) a period of levetiracetam administration exceeding 30 days, and d) contraindications for MRI.

Clinical characteristics

The information on age, sex, education time, Karnofsky performance score, extent of tumor resection, histopathology, isocitrate dehydrogenase mutation and chromosome 1p/19q co-deletion, history of preoperative seizures, and performances during seizure onset were acquired from inpatient records. Follow-up information was obtained by telephone interviews at 6 months after tumor resections.

MRI acquisition

We used a 3-T MR scanner (MAGNETOM Prisma, Siemens, Erlangen, Germany)

to acquire MR data. The T2 sequence with fluid-attenuated inversion recovery (FLAIR) (echo time [TE], 87 ms; repetition time [TR], 3200 ms; field of view [FOV], 220 mm * 220 mm; fractional anisotropy [FA], 150°; voxel size, 0.9 mm * 0.9 mm * 5 mm; slice number, 25). Moreover, we used rs-fMRI sequence to acquire functional image data (TE, 30 ms; TR, 2000 ms; FOV, 220 mm * 220 mm; FA, 75°; voxel size, 3.0 mm * 3.0 mm * 5.0 mm; slice number, 30; and acquisition time, 8 min.

All patients were scanned within 3 days before surgery.

Pipeline of rs-MRI preprocessing

A software, Graph Theoretical Network Analysis (https://www.nitrc.org/projects/gretna), [12, 13] was used to process rs-fMRI data. The pipeline was the same as in the previous study[10] and is shown in the supplementary materials.

Regions of tumor invasion

Each glioma was segmented in its individual space based on the region with hyper-intensity in T2-FLAIR images. The extent of glioma infiltration was manually and independently determined by two neuroradiologists. If the determined regions varied more than 5%, the final decision was made by a third neuroradiologist who had over 20 years' clinical experience. Subsequently, all tumor masks were normalized into the standard space of the Montreal Neurological Institute template by using SPM8 software (http://www.fil.ion.ucl.ac.uk/spm/software/spm8).

Regions of interest

Regions of interest (ROIs) were generated from "brainnetome atlas" (http://www.brainnetome.org/).[14] This open-access atlas comprises 246 brain regions, to acquire matrices of FC. Four sub-templates were extracted, which were sensorimotor, language, left executive control, and visual networks. The ROIs in these networks

invaded by glioma were excluded. Hence, the inaccurate effect of registration was reduced as much as possible. Detailed information on each ROI in presented in Tables S1 to S4.

Network construction

The mean time series between each two ROIs were compared using Pearson correlation, and subsequently the FC matrices were constructed. Consequently, we obtained four different FC matrices.

Graph theoretical measurement

Topological properties of the four sub-networks were analyzed using graph theory measurement, which included global properties (the shortest path length, global efficiency, local efficiency, clustering coefficient, transitivity, and vulnerability), nodal properties (nodal efficiency, nodal local efficiency, nodal clustering coefficient, degree centrality), and small-worldness. [10, 15, 16] The details of properties were shown in part 2 of the supplementary materials. All matrices were absolutized and binarized to further analyze the topological properties.

Statistical analyses

Epidemiology characteristics were compared among the GRE, non-GRE, and healthy groups by using Student's *t*-test, Mann-Whitney U test, Chi-squared test, Fisher's exact test, and one-way analysis of variance (one-way ANOVA) based on categories of data. All data were tested to ensure whether they were normal/Gaussian distribution. If a group of data did not exhibit a normal distribution, a student's t test or one-way ANOVA test was applied with a non-parametric equivalent.

The differences in FC of the four sub-networks were generated from comparisons between the patient and healthy groups using Student's *t*-test. Moreover, false discovery rate (FDR) was applied to correct the generated results. To found differences in

topological properties, we used a series of sparsity thresholds (from 0.17 to 0.33, interval 0.01) consistent with a previously study.[4] For each subject, topological properties were generated according to sparsity. Each property was first analyzed using one-way ANOVA test. Subsequently, post-hoc pairwise comparisons were performed on the generated results in global and nodal properties with least significant difference (LSD) test. A significant p-value was lower than 0.05.

Data availability statement

Anonymized data will be made available on request.

Results

Demographic characteristics

Fifty-six patients met the inclusion criteria, and four patients were excluded, as their periods of anti-epileptic drug use were longer than 30 days. According to the history of preoperative GRE onset, 28 patients were divided into the GRE group (male, n = 11) and the others into the non-GRE group (male, n = 14, Table 1). All patients were right-handed according to the assessments by the Edinburgh Handedness Inventory test, and their epilepsy onset performance was considered as a secondary generalized epilepsy. Our postoperative follow-up showed that, all patients achieved Engel class I at 6 months after tumor resection. Based on the sample size of each group, 28 healthy participants were finally recruited after matching age, sex, and education level (14 males; all right-handed).

No differences in Karnofsky performance score ($p = 0.12$, Mann-Whitney U test), ratio of histopathology ($p = 0.73$), isocitrate dehydrogenase mutation ($p = 0.59$, Chi-squared test), or chromosome 1p/19q co-deletion ($p = 0.53$, Chi-squared test) were observed between the GRE and non-GRE groups. Moreover, no difference in tumor volume ($p = 0.75$) was found between the GRE (52.38 ± 7.06 mL) and non-GRE groups

(49.65 ± 4.98 mL).

Functional connectivity differences

Our results revealed no differences in FC of the four sub-networks (sensorimotor, visual, language, and left executive control networks) among the three groups after FDR correction.

Differences in global topological properties

In the language network, the clustering coefficient ($p = 0.0070$), global efficiency (p < 0.0001), local efficiency ($p = 0.0045$), shortest path length ($p < 0.0001$), transitivity (p = 0.0002), and vulnerability ($p = 0.0499$) were different among the three groups, as determined using one-way ANOVA (Table S5).

Post-hoc analysis with the LSD test (Fig. 1) revealed that the non-GRE group exhibited weaker global efficiency (0.502 ± 0.005) than the GRE (0.522 ± 0.003, $p < 0.0001$) and control groups (0.525 ± 0.002, $p < 0.0001$). Moreover, the non-GRE group showed greater local efficiency (0.465 ± 0.012) than the GRE (0.430 ± 0.010, $p = 0.0016$) and control groups (0.437 ± 0.010, $p = 0.0165$). The non-GRE group exhibited a longer shortest path length (2.089 ± 0.015) than the GRE (2.023 ± 0.008, $p < 0.0001$) and control groups (2.015 ± 0.006, $p < 0.0001$). Furthermore, the non-GRE group showed a greater clustering coefficient (0.577 ± 0.002) than the GRE (0.325 ± 0.010, $p = 0.0020$) and control groups (0.334 ± 0.009, $p = 0.0348$). No differences in global efficiency ($p = 0.5939$), local efficiency (p = 0.4157), shortest path length ($p = 0.6025$), or clustering coefficient ($p = 0.9079$) were found between the GRE and control groups.

Post-hoc analysis using the LSD test (Fig. 2) revealed that the non-GRE group had greater transitivity (0.372 ± 0.015) than the GRE (0.316 ± 0.009, $p = 0.0011$) and control groups (0.315 ± 0.007, $p = 0.0009$). Moreover, the non-GRE group had more severe vulnerability (0.071 ± 0.005) than the GRE (0.060 ± 0.003, $p = 0.0371$) and

control groups (0.060 ± 0.003, $p = 0.0371$). No differences in transitivity ($p = 0.9524$) or vulnerability ($p = 0.9999$) were found between the GRE and control groups.

No differences in global topological properties were found in the other three sub-networks (sensorimotor, visual, and left executive networks).

Differences in small-worldness properties

In the language network, the value of lambda ($p < 0.0001$) differed among the groups, as determined using one-way ANOVA (Table S5 and Fig. 3). No differences in the values of gamma ($p = 0.4822$) or sigma ($p = 0.5176$) were found among the three groups. Post-hoc analysis using the LSD test showed that the non-GRE group exhibited a higher value of lambda (1.043 ± 0.006) than the GRE (1.016 ± 0.003, p < 0.0001) and control groups (1.014 ± 0.002, p < 0.0001). No difference in the value of lambda ($p = 0.5969$) was found between the GRE and control groups.

No significant alterations in small-worldness (gamma, lambda, and sigma) in the other three sub-networks (sensorimotor, visual, and left executive networks) were found among the three groups.

Differences in nodal topological properties

One-way ANOVA revealed two nodes in the right hemisphere that had differing nodal efficiencies among the three groups in the language network (Table S6 and Fig. 4): rostroventral BA 39 (A39rv_R, $p = 0.0002$) and rostral BA 45 (A45r_R, $p = 0.0060$). Regarding A39rv_R, the non-GRE group had weaker nodal efficiency (0.483 ± 0.019) than the GRE (0.558 ± 0.012, $p = 0.0014$) and control groups (0.560 ± 0.012, $p = 0.0010$) after post-hoc analysis. Similarly, regarding A45r_R, the non-GRE group showed weaker nodal efficiency (0.467 ± 0.017) than the GRE (0.520 ± 0.008, $p = 0.0207$) and control groups (0.523 ± 0.013, $p = 0.0129$) after post-hoc analysis. No differences in nodal efficiency of these two nodes were found between the GRE and control groups

(A39rv_R, $p = 0.8903$ and A45r_R, $p = 0.8693$).

Among the three groups, some differences in nodal local efficiency, degree centrality, and nodal clustering coefficient were found in caudal BA 40 (A40c_L, $p = 0.0061$), ventral BA 44 (A44v_L, $p = 0.0007$), and rostral BA 22 (A22r_L, $p = 0.0097$) in the left hemisphere, as determined using one-way ANOVA (Tables S7–S9 and Fig. 5). Regarding A40c_L, the GRE group exhibited weaker nodal local efficiency (0.301 ± 0.032) than the non-GRE (0.455 ± 0.039, p = 0.0102) and control groups (0.437 ± 0.037, $p = 0.0280$) after post-hoc analysis. Regarding A44v_L, the non-GRE group exhibited greater degree centrality (5.821 ± 0.349) than the GRE (4.750 ± 0.250, $p = 0.0282$) and control groups (4.071 ± 0.262, $p = 0.0005$) after post-hoc analysis. With regard to A22r_L, the non-GRE group exhibited a greater nodal clustering coefficient (0.386 ± 0.048) than the GRE (0.229 ± 0.037, $p = 0.0150$) and control groups (0.250 ± 0.027, $p = 0.0433$) after post-hoc analysis.

No significant alterations in nodal topological properties in the other three sub-networks (sensorimotor, visual, and left executive networks) were found among the three groups.

Discussion

In this study, we investigated alterations in functional neural networks induced by right temporal GRE. Our findings indicated that GRE and right-temporal DLGGs resulted in altered language networks. Although the altered network differed from the left-temporal GRE change (visual network), the trend of right-temporal DLGGs and GRE-induced functional network change was the same as that of the left. That is, the GRE-induced functional network change was found to be opposite of that induced by DLGG.

Global efficiency and shortest path length reflect the ability and cost of conveying

information, respectively.[17] In our findings, right-temporal glioma decreased global efficiency and increased the shortest path length of the language network in the non-GRE group. These changes were related to a neural pathway disruption caused by glioma infiltration. Indeed, the main language network is located in the left hemisphere in right-handed people.[18] The fMRI results suggested that when the left language network was damaged, functional compensation occurred in the cortex of the right hemisphere corresponding to the left language regions.[19] These findings indicate that parts of the language network are located on the right and cooperated with the left network to accomplish language tasks.[20, 21] Consequently, if the right-sided language network was damaged by glioma, the global efficiency of the whole language network was decreased in the non-GRE group. Indeed, compared with the GRE and healthy groups, the clustering coefficient and local efficiency in the non-GRE group was increased. These alterations reflect the increase in the number of functional connections, but this does not mean that the pathway between two nodes was shortened or the ability to convey information was increased. Moreover, the right-temporal glioma failed to disrupt the main language network (left hemisphere) and was unable to further induce language deficits. Hence, the language network did not drastically reorganize in the non-GRE group. Therefore, we infer that the global decline in the efficiency of the language network was the result of the damage caused by glioma, which increased the burden of the residual language network.

However, in the GRE group, global efficiency did not differ from that in the healthy group. Why was there no decrease in global efficiency in the GRE group? These alterations were associated with GRE-induced network reorganization, but they did not indicate that GRE facilitated recovery of the language network. Unlike primary epilepsy with a long and frequent onset history, the period from onset of GRE to glioma

diagnosis and tumor resection is short. Hence, cortical sclerosis,[22] gray-matter atrophy,[23] and cortical hypo-metabolism[24] did not occur in patients with GRE. Conversely, we found that the path length in the GRE group was shorter than that in the non-GRE group. The GRE shortens pathways to decrease system response time, which facilitates the rapid spread of local epileptic discharges.[25, 26] Thus, we concluded that the mechanism of GRE altering the language network was as follows: the right-temporal glioma first disrupted the right-sided language network and decreased global efficiency; then, accompanied by the GRE, the residual network was reorganized with increased global efficiency.

Vulnerability represents the degree of global efficiency alteration when a node is replaced and reflects whether a neural network is stable.[27] We observed that the vulnerability in the non-GRE group was higher than that in the GRE and control groups. This finding indicated that the right-temporal glioma rendered the residual language network vulnerable, and to maintain the residual language function normally, none of the nodes could be further broken or replaced. Simultaneously, this finding verified that GRE facilitated network reorganization to regain stability.

Similar results were found for nodal properties. The decreasing nodal efficiency of the right inferior frontal and supramarginal gyri in the non-GRE group showed that the glioma damaged the original right language network and affected the two important nodes needed to process language information. Simultaneously, the left nodes in the language network had to increase the degree centrality (Broca area), nodal local efficiency (Wernicke area), and nodal clustering coefficient (in the left temporal lobe) to maintain language functions. However, influenced by both glioma and GRE, the alterations in these nodal properties were alleviated in the GRE group.

Gliomas located in different hemispheres will affect different neural networks, but

the GRE and glioma itself induced the same network alterations. Based on our findings, the right-temporal glioma and GRE affected the language network rather than the visual network, which was shown to be affected by left-temporal glioma and GRE in a previous study. [10]We thought that the different affected networks were related to the dominant hemisphere. When glioma is located in the left hemisphere, the main language network is damaged and residual language network reorganization occurs, whether caused by the glioma itself or GRE. Hence, no differences in language network alterations were found between the GRE and non-GRE groups in the previous study. Regarding the right-temporal glioma, the main language network was not affected. Therefore, the alterations in the language network caused by the glioma itself and the GRE were significantly different. A common trend is that the changes in the neural networks caused by glioma itself or GRE are converse, regardless whether the tumor is located on the left or right hemisphere.

Limitations

The phenomenon that levetiracetam normalized FC was found in patients with primary epilepsy who took levetiracetam over 3 months.[28] In our GRE group, all patients indeed took levetiracetam, but the period of administration was short (not longer than 15 days). To our knowledge, no study has revealed whether taking levetiracetam in a short period would alter topological properties. Hence, we could not determine whether alterations of topological properties in patients with GRE tended to normalize due to levetiracetam administration. In the future, we will enroll relevant patients to investigate whether taking levetiracetam for a short period can induce alterations of the functional network in patients with GRE and validate the findings in this study.

Conclusions

Different tumor locations alter different neural networks. Temporal-lobe gliomas in the right hemisphere altered the language network. Alterations in the language network caused by GRE were opposite to those caused by glioma itself. Our findings provide a novel insight into the GRE impact and improve our understanding of alterations in functional neural networks in patients with glioma. In addition, under the premise of protecting the language function, postoperative epileptic onset might be effectively controlled by electrical cauterizing the pia mater in the language network in patients with GRE.

Acknowledgments

Thanks to Dr. Meng Lanxi for imaging data acquisition.

Disclosure

All authors do not have any conflict of interest.

Funding information

This study was supported by funds from the Public Welfare Development and Reform Pilot Project of Beijing Medical Research Institute (PXM2019_026280_000008), Beijing Municipal Natural Science Foundation (No. 7202021), National Natural Science Foundation of China (No. 82001777), National Key Research and Development Program of China (2018YFC0115604), and Brain Tumor Precision Diagnosis and Treatment and Translational Medicine Innovation Unit, Chinese Academy of Medical Sciences (2019-I2M-5-021).

References

1. Liang S, Fan X, Zhao M, Shan X, Li W, Ding P, et al. Clinical practice guidelines for the diagnosis and treatment of adult diffuse glioma-related epilepsy. Cancer Med. 2019 Aug;8(10):4527-35.
2. Li Y, Shan X, Wu Z, Wang Y, Ling M, Fan X. IDH1 mutation is associated with a higher preoperative seizure incidence in low-grade glioma: A systematic review and meta-analysis. Seizure. 2018 Feb;55:76-82.
3. Shan X, Fan X, Liu X, Zhao Z, Wang Y, Jiang T. Clinical characteristics associated with postoperative seizure control in adult low-grade gliomas: a systematic review and meta-analysis. Neuro-oncology. 2018 Feb 19;20(3):324-31.
4. Ji GJ, Yu Y, Miao HH, Wang ZJ, Tang YL, Liao W. Decreased Network Efficiency in Benign Epilepsy with Centrotemporal Spikes. Radiology. 2017 Apr;283(1):186-94.
5. Li Q, Chen Y, Wei Y, Chen S, Ma L, He Z, et al. Functional Network Connectivity Patterns between Idiopathic Generalized Epilepsy with Myoclonic and Absence Seizures. Front Comput Neurosci. 2017;11:38.
6. Bernhardt BC, Chen Z, He Y, Evans AC, Bernasconi N. Graph-theoretical analysis reveals disrupted small-world organization of cortical thickness correlation networks in temporal lobe epilepsy. Cereb Cortex. 2011 Sep;21(9):2147-57.
7. He Y, Wang J, Wang L, Chen ZJ, Yan C, Yang H, et al. Uncovering intrinsic modular organization of spontaneous brain activity in humans. PLoS One. 2009;4(4):e5226.
8. Englot DJ, Hinkley LB, Kort NS, Imber BS, Mizuiri D, Honma SM, et al. Global and regional functional connectivity maps of neural oscillations in focal epilepsy. Brain. 2015 Aug;138(Pt 8):2249-62.
9. Englot DJ, Gonzalez HFJ, Reynolds BB, Konrad PE, Jacobs ML, Gore JC, et al. Relating structural and functional brainstem connectivity to disease measures in epilepsy. Neurology. 2018 Jul 3;91(1):e67-e77.
10. Fang S, Zhou C, Fan X, Jiang T, Wang Y. Epilepsy-Related Brain Network Alterations in Patients With Temporal Lobe Glioma in the Left Hemisphere. Front Neurol. 2020;11:684.
11. Louis DN, Perry A, Reifenberger G, von Deimling A, Figarella-Branger D, Cavenee WK, et al. The 2016 World Health Organization Classification of Tumors of the Central Nervous System: a summary. Acta Neuropathol. 2016 Jun;131(6):803-20.
12. Wang J, Wang X, Xia M, Liao X, Evans A, He Y. Corrigendum: GRETNA: a graph theoretical network analysis toolbox for imaging connectomics. Front Hum Neurosci. 2015;9:458.
13. Wang J, Wang X, Xia M, Liao X, Evans A, He Y. GRETNA: a graph theoretical network analysis toolbox for imaging connectomics. Front Hum Neurosci. 2015;9:386.
14. Fan L, Li H, Zhuo J, Zhang Y, Wang J, Chen L, et al. The Human Brainnetome Atlas: A New Brain Atlas Based on Connectional Architecture. Cereb Cortex. 2016 Aug;26(8):3508-26.
15. Qi S, Mu YF, Cui LB, Zhang J, Guo F, Tan QR, et al. Anomalous gray matter structural networks in recent onset post-traumatic stress disorder. Brain Imaging Behav. 2018 Apr;12(2):390-401.
16. Caeyenberghs K, Powell HW, Thomas RH, Brindley L, Church C, Evans J, et al. Hyperconnectivity in juvenile myoclonic epilepsy: a network analysis. Neuroimage Clin. 2015;7:98-104.
17. Zhao T, Cao M, Niu H, Zuo XN, Evans A, He Y, et al. Age-related changes in the topological organization of the white matter structural connectome across the human

lifespan. Hum Brain Mapp. 2015 Oct;36(10):3777-92.
18. Kundu B, Rolston JD, Grandhi R. Mapping language dominance through the lens of the Wada test. Neurosurg Focus. 2019 Sep 1;47(3):E5.
19. Deng X, Zhang Y, Xu L, Wang B, Wang S, Wu J, et al. Comparison of language cortex reorganization patterns between cerebral arteriovenous malformations and gliomas: a functional MRI study. J Neurosurg. 2015 May;122(5):996-1003.
20. Gebska-Kosla K, Bryszewski B, Jaskolski DJ, Fortuniak J, Niewodniczy M, Stefanczyk L, et al. Reorganization of language centers in patients with brain tumors located in eloquent speech areas - A pre- and postoperative preliminary fMRI study. Neurol Neurochir Pol. 2017 Sep - Oct;51(5):403-10.
21. Gunal V, Savardekar AR, Devi BI, Bharath RD. Preoperative functional magnetic resonance imaging in patients undergoing surgery for tumors around left (dominant) inferior frontal gyrus region. Surg Neurol Int. 2018;9:126.
22. Aparicio J, Carreno M, Bargallo N, Setoain X, Rubi S, Rumia J, et al. Combined (18)F-FDG-PET and diffusion tensor imaging in mesial temporal lobe epilepsy with hippocampal sclerosis. Neuroimage Clin. 2016;12:976-89.
23. Caciagli L, Bernasconi A, Wiebe S, Koepp MJ, Bernasconi N, Bernhardt BC. A meta-analysis on progressive atrophy in intractable temporal lobe epilepsy: Time is brain? Neurology. 2017 Aug 1;89(5):506-16.
24. Celiker Uslu S, Yuksel B, Tekin B, Sariahmetoglu H, Atakli D. Cognitive impairment and drug responsiveness in mesial temporal lobe epilepsy. Epilepsy Behav. 2019 Jan;90:162-7.
25. Ponten SC, Bartolomei F, Stam CJ. Small-world networks and epilepsy: graph theoretical analysis of intracerebrally recorded mesial temporal lobe seizures. Clin Neurophysiol. 2007 Apr;118(4):918-27.
26. Dyhrfjeld-Johnsen J, Santhakumar V, Morgan RJ, Huerta R, Tsimring L, Soltesz I. Topological determinants of epileptogenesis in large-scale structural and functional models of the dentate gyrus derived from experimental data. J Neurophysiol. 2007 Feb;97(2):1566-87.
27. Latora V, Marchiori M. Vulnerability and protection of infrastructure networks. Phys Rev E Stat Nonlin Soft Matter Phys. 2005 Jan;71(1 Pt 2):015103.
28. Pang XM, Liang XL, Zhou X, Liu JP, Zhang Z, Zheng JO. Alterations in intra- and internetwork functional connectivity associated with levetiracetam treatment in temporal lobe epilepsy. Neurol Sci. 2020 Mar 9.

Table 1

Table 1. Demographic and Clinical Characteristics of patient groups

Demographic and Clinical Characteristics	GRE (n = 28)	non-GRE (n = 28)	Healthy (n = 28)	p value
**Gender**				
Male	11	14	14	0.65
Female	17	14	14	
**Age (y) ***	41.4 ± 2.3	46.0 ± 2.2	39.7 ± 1.7	0.09
**Handness**				
Right	28	28	28	-
Left	0	0	0	
**KPS score (preoperative)**				
100	24	26	28	0.12
90~100	4	2	0	
**Education level (y)***	13.0 ± 0.6	12.0 ± 0.6	13.4 ± 0.6	0.16
**Histopathology**				
Astrocytoma	8	6	-	0.73
Oligodendroglioma	4	7	-	
Anaplastic Astrocytoma	13	13	-	
Anaplastic Oligodendroglioma	3	2	-	
**IDH status**				
Mutation	11	13		0.59
Wild-type	17	15		
**Chromosome 1p/19q status**				
Codeletion	6	8		0.53
Non-codeletion	22	20		
**Tumor volume (mL)***	52.38 ± 7.06	49.65 ± 4.98	-	0.85
**Onset age (y)***	41.1 ± 2.1	-	-	-
**Frequency before diagnosis**				
Low (only once)	23			
Medium (2~3 times)	3			
High (>3 times)	2			
**Preoperative anti-epileptic drugs**				
Levetiracetam (0.5g, twice a day)	28			-
**Postoperative epileptic control**				
Engel Class I	28			-

* Values are means ± standard error of mean.

Using Mann-Whitney U test to compare the difference of Karnofsky performance status between GRE and non-GRE groups.

Using student t test via non-parametric equivalent to compare the difference of tumor volume between GRE and non-GRE groups.

Using one-way ANOVA test to compare the difference of age between patients groups and healthy group.

Using one-way ANOVA test via non-parametric equivalent to compare the difference of education level between patients groups and healthy group.

Using to Chi-square test to compare the differences of gender, tumor location, and IDH status between GRE and non-GRE groups.

KPS = Karnofsky performance status

Figure legends

Figure 1. Results of alterations in global topological properties when gliomas grew in the right temporal lobe. The grp GRE = group of patients with glioma-related epilepsy. The grp non-GRE = group of patients without glioma-related epilepsy. The grp healthy = group of healthy participants.

Figure 2. Results of alterations in transitivity and vulnerability when gliomas grew in the right temporal lobe. The grp GRE = group of patients with glioma-related epilepsy. The grp non-GRE = group of patients without glioma-related epilepsy. The grp healthy = group of healthy participants.

Figure 3. Results of alterations in small-worldness when gliomas grew in the right temporal lobe. The grp GRE = group of patients with glioma-related epilepsy. The grp non-GRE = group of patients without glioma-related epilepsy. The grp healthy = group of healthy participants.

Figure 4. Results of alterations in nodal properties of right nodes in the language network when gliomas grew in the right temporal lobe. The grp GRE = group of patients with glioma-related epilepsy. The grp non-GRE = group of patients without glioma-related epilepsy. The grp healthy = group of healthy participants.

Figure 5. Results of alterations in nodal properties of left nodes in the language network when gliomas grew in the right temporal lobe. The grp GRE = group of patients with glioma-related epilepsy. The grp non-GRE = group of patients without glioma-related epilepsy. The grp healthy = group of healthy participants.

Supplementary materials

Part 1. Processing pipeline of rs-fMRI data.

The rs-fMRI data were processed as follows: a) transformation to a NIFTI file, b) removal of the first 5 time points, c) slice timing, d) realignment, e) normalization (normalized to EPI template [1]), f) smoothing (full width half maximum = 4 mm), g) temporal detrending (linear detrending), h) covariance regressing (white matter signal: with WMMask_3mm; CSF signal: with CSFMask_3mm; head motion: Friston – 24 parameters), i) temporal filtering (0.01–0.08 Hz), and j) scrubbing (linear interpolation, subsequent time point number = 2, FD threshold = 0.5, and previous time point number = 1,).

Part 2. Information of Topological Properties

Clustering coefficient

Cluster coefficient represents the possibility that the neighbors of node *j* can interact with other nodes, and meant clustering degree of functional network. The formula was as follows:

$$Ci = \frac{2ej}{kj(kj-1)}$$

$C_{j,}$ cluster coefficient of node *j*; k_{j}, the number of probable edges connecting to other nodes; $e_{j,}$ the number of actual edges connecting to other nodes.

Global efficiency

Global efficiency meant the ability of information transmission at global level. The formula was as follows: (reference to supplementary materials of Ji G et al, Radiology, 2017 [2]):

$$E_{\text{glob}} = \frac{1}{N(N-1)} \sum_{i,j \in V, i \neq j} \frac{1}{l_{ij}}.$$

E_{glob}, global efficiency of whole functional network; N, the number of actual edges connecting node *i* to other nodes in the whole network; $l_{i,\ k}$, the shortest path length between the nodes j and k.

Shortest path length

The shortest path length meant the minimum number of passing edges for information conduction between each two nodes. It describes the optimal pathway for information transmission at global level. The calculation formula was as follows:

$$L = \frac{1}{N(N-1)} \sum_{i,j \in V, i \neq j} l_{ij}$$

L, shortest path length of network; N, the number of actual edges connecting the node *i* to another node in the whole network; $l_{i,\ k}$, the shortest path length between the node j and node k.

Local efficiency

Local efficiency represents the ability of information conduction in local network. The calculation formula as follows:

$$E(i) = \frac{1}{N_{G_i}(N_{G_i} - 1)} \sum_{j \neq k \in G_i} \frac{1}{l_{j,k}}$$

E_i, local efficiency of local network; N_{G_i}, the number of actual edges connecting node *i* to other nodes in the local network; $l_{i,\ k}$, the shortest path length between the nodes j and k.

Nodal efficiency

Nodal efficiency represents the ability of information transmission of the node i. The calculation formula as follows:

$$E_{nodal} = \frac{1}{(N-1)} \sum_{i,j \in V, i \neq j} \frac{1}{l_{ij}}$$

E_{nodal}, nodal efficiency of whole functional network; N, the number of actual edges connecting node *i* to other nodes in the whole functional network; $l_{i,k}$, the shortest path length between the nodes j and k.

Small-worldness properties

Small-worldness properties (gamma, lambda, and sigma) mean the efficiency of information transmission. Gamma (γ) = C_{real}/C_{random} >> 1 (C = cluster coefficient). Lambda (λ) = L_{real}/L_{random} ~ 1 (L = shortest path length). And Sigma (σ) = γ/λ > 1. [3, 4] If a network has a high value of sigma, the ability of information transmission will be strong.

Part 3. Supplemental Tables

Table S1. Montreal Neurological Institute (MNI) locations of 22 nodes in the language network

Regions of interesting	Modified Cyto-architectonic	Left hemisphere			Right hemisphere		
		X	Y	Z	X	Y	Z
A45c_L(R)	caudal BA 45	-53	23	11	54	24	12
A45r_L(R)	rostral BA 45	-49	36	-3	51	36	-1
A44op _L(R)	opercula BA 44	-39	23	4	42	22	3
A44v _L(R)	ventral BA 44	-52	13	6	54	14	11
A12/47l _L(R)	lateral BA 12/47	-41	32	-9	42	31	-9
A4hf _L	Area BA 4(head and face region)	-8	-38	58	-	-	-
A41/42_L	Area BA 41/42	-54	-32	12	-	-	-
A22c _L	caudal BA 22	-62	-33	7	-	-	-
A22r _L	rostral BA 22	-55	-3	-10	-	-	-
A21c_L	caudal BA 21	-65	-30	-12	-	-	-
aSTS_L	anterior superior temporal sulcus	-58	-20	-9	-	-	-
rpSTS_L	rostroposterior superior temporal sulcus	-54	-40	4	-	-	-
cpSTS_L	caudoposterior superior temporal sulcus	-52	-50	11	-	-	-
A40c_L(R)	caudal area 40(PFm)	-56	-49	38	57	-44	38
A39rv_L(R)	rostroventral area 39(PGa)	-47	-65	26	53	-54	25

*BA = Brodmann area.

Table S2. Montreal Neurological Institute (MNI) locations of 22 nodes in the visual network.

Regions of interesting	Modified Cyto-architectonic	Left hemisphere			Right hemisphere		
		X	Y	Z	X	Y	Z
cLinG_L(R)	caudal lingual gyrus	-11	-82	-11	10	-85	-9
rCunG_L(R)	rostral cuneus gyrus	-5	-81	10	7	-76	11
cCunG_L(R)	caudal cuneus gyrus	-6	94	1	8	-90	12
rLinG_L(R)	rostral lingual gyrus	-17	-60	-6	18	-60	-7
vmPOS_L(R)	ventromedial parietal occipital sulcus	-13	-68	12	15	-63	12
mOccG_L(R)	middle occipital gyrus	-31	-89	11	34	-86	11
V5/MT+_L(R)	area V5/MT+	-46	-74	3	48	-70	-1
OPC_L(R)	occipital polar cortex	-18	-99	2	22	-97	4
iOccG_L(R)	inferior occipital gyrus	-30	-88	-12	32	-85	-12
msOccG_L(R)	medial superior occipital gyrus	-11	-88	31	16	-85	34
lsOccG_L(R)	lateral superior occipital gyrus	-22	-77	36	29	-75	36

*BA = Brodmann area.

Table S3. Montreal Neurological Institute (MNI) locations of 20 nodes in the left executive control network.

Regions of interesting	Modified Cyto-architectonic	Left hemisphere		
		X	Y	Z
A8dl_L	dorsolateral area BA 8	-18	24	53
A9m_L	medial area BA 9	-5	36	38
A9/46d_L	dorsal area BA 9/46	-27	43	31
IFJ_L	inferior frontal junction	-42	13	36
A46_L	area BA 46	-28	56	12
A9/46v_L	ventral area BA 9/46	-41	41	16
A8vl_L	ventrolateral area BA 8	-33	23	45
A6vl_L	ventrolateral area BA 6	-32	4	55
A10l_L	ventrolateral area BA 10	-26	60	-6
IFS_L	inferior frontal sulcus	-47	32	14
A45r_L	rostral area BA 45	49	36	-3
A12/47l_L	lateral area BA 12/47	-41	32	-9
A21c_L	caudal area BA 21	-65	-30	-12
A37vl_L	ventrolateral area BA 37	-55	-60	-6
A20cl_L	caudal-lateral of area BA 20	-59	-42	-16
A7ip_L	intraparietal area BA 7 (hIP3)	-27	-59	54
A39c_L	caudal area 39 (PGp)	-34	-80	29
A39rd_L	rostral dorsal area BA 39 (Hip3)	-38	-61	46
A40c_L	caudal area BA 40 (PFm)	-56	-49	38
A39rv_L	rostroventral area BA 39 (PGa)	-47	-65	26

*BA = Brodmann area.

1 **Table S4. Montreal Neurological Institute (MNI) locations of 22 nodes in the**
2 **sensorimotor network**

Regions of interesting	Modified Cyto-architectonic	Left hemisphere			Right hemisphere		
		X	Y	Z	X	Y	Z
A6m_L(R)	Medial area BA 6	5	36	38	6	38	35
A4hf_L(R)	Area BA 4 (head and face)	-49	-8	39	55	-2	33
A4ul_L(R)	Area BA 4 (upper limb)	-26	-25	63	34	-19	59
A4t_L(R)	Area BA 4 (trunk)	-13	-20	73	15	-22	71
A4tl_L(R)	Area BA 4 (tongue and larynx)	-52	0	8	54	4	9
A1_2_3ll_L(R)	Area BA 1/2/3 (lower limb)	-8	-38	58	10	-34	54
A4ll_L(R)	Area BA 4 (lower limb)	-4	-23	61	5	-21	61
A1_2_3ulhf_L(R)	Area BA 1/2/3 (upper limb and face)	-50	-16	43	50	-14	44
A1_2_3tonIa_L(R)	Area BA 1/2/3 (tongue and larynx)	-56	-14	16	56	-10	15
A2_L(R)	Area BA 2	-46	-30	50	48	-24	48
A1_2_3tru_L(R)	Area BA 1/2/3 (trunk)	-21	-35	68	20	-33	69

3 *BA = Brodmann area.

4

Table S5. Global Topological properties compared between the patient and healthy groups

	GRE group	non-GRE group	Health group	One-way ANOVA (p value)	GRE vs non-GRE (p value)	GRE vs Health (p value)	non-GRE vs n Health (p value)
Global efficiency	0.522 ± 0.003	0.502 ± 0.005	0.525 ± 0.002	< 0.0001	< 0.0001	0.5939	< 0.0001
Local efficiency	0.430 ± 0.010	0.465 ± 0.012	0.437 ± 0.010	0.0045	0.0016	0.4157	0.0165
Shortest path length	2.023 ± 0.008	2.089 ± 0.015	2.015 ± 0.006	< 0.0001	< 0.0001	0.6025	< 0.0001
Clustering coefficient	0.325 ± 0.010	0.577 ± 0.002	0.334 ± 0.009	0.0070	0.0020	0.9079	0.0348
Gamma	1.241 ± 0.035	1.289 ± 0.034	1.278 ± 0.030	0.4822	-	-	-
Lambda	1.016 ± 0.003	1.043 ± 0.006	1.014 ± 0.002	< 0.0001	< 0.0001	0.5969	< 0.0001
Sigma	1.221 ± 0.033	1.232 ± 0.032	1.259 ± 0.029	0.5176	-	-	-
Transitivity	0.316 ± 0.009	0.372 ± 0.015	0.315 ± 0.007	0.0002	0.0011	0.9524	0.0009
Vulnerability	0.060 ± 0.003	0.071 ± 0.005	0.060 ± 0.003	0.0499	0.0371	0.9999	0.0371

* The global properties were calculated with one-way ANOVA test. If the results one-way ANOVA were significance, post-hoc analysis with least significant difference was subsequently applied.

Table S6. Nodal efficiency compared between the patients and healthy groups

Node name	GRE group	non-GRE group	Health group	One-way ANOVA (p value)	GRE vs non-GRE (p value)	GRE vs Health (p value)	non-GRE vs n Health (p value)
A45c_L	0.543 ± 0.013	0.497 ± 0.024	0.535 ± 0.012	0.1396	-	-	-
A45c_R	0.518 ± 0.015	0.462 ± 0.025	0.535 ± 0.014	0.0180	-	-	-
A45r_L	0.540 ± 0.015	0.506 ± 0.017	0.517 ± 0.016	0.3018	-	-	-
A45r_R	0.520 ± 0.008	0.467 ± 0.017	0.523 ± 0.013	0.0060	0.0207	0.8693	0.0129
A44op _L	0.505 ± 0.013	0.502 ± 0.016	0.502 ± 0.022	0.9891	-	-	-
A44op _R	0.525 ± 0.019	0.477 ± 0.026	0.482 ± 0.018	0.2202	-	-	-
A44v _L	0.540 ± 0.010	0.560 ± 0.014	0.522 ± 0.010	0.0677	-	-	-
A44v _R	0.497 ± 0.014	0.510 ± 0.020	0.510 ± 0.022	0.8612	-	-	-
A12/47l _L	0.533 ± 0.013	0.480 ± 0.029	0.508 ± 0.017	0.1941	-	-	-
A12/47l _R	0.517 ± 0.014	0.512 ± 0.012	0.511 ± 0.016	0.9520	-	-	-
A4hf _L	0.480 ± 0.018	0.575 ± 0.015	0.474 ± 0.020	0.1507	-	-	-
A41/42_L	0.514 ± 0.017	0.503 ± 0.015	0.541 ± 0.016	0.2208	-	-	-
A22c _L	0.522 ± 0.012	0.508 ± 0.013	0.513 ± 0.016	0.7776	-	-	-
A22r _L	0.496 ± 0.024	0.497 ± 0.020	0.540 ± 0.010	0.1700	-	-	-
A21c_L	0.532 ± 0.015	0.529 ± 0.016	0.535 ± 0.012	0.9579	-	-	-
aSTS_L	0.515 ± 0.016	0.508 ± 0.020	0.540 ± 0.015	0.3948	-	-	-
rpSTS_L	0.546 ± 0.014	0.528 ± 0.020	0.538 ± 0.012	0.7284	-	-	-

cpSTS_L	0.490 ± 0.022	0.513 ± 0.024	0.522 ± 0.017	0.5430	-	-	-
A40c_L	0.538 ± 0.015	0.529 ± 0.021	0.562 ± 0.011	0.3294	-	-	-
A40c_R	0.512 ± 0.024	0.493 ± 0.022	0.540 ± 0.014	0.2683	-	-	-
A39rv_L	0.547 ± 0.014	0.541 ± 0.016	0.534 ± 0.016	0.8350	-	-	-
A39rv_R	0.558 ± 0.012	0.483 ± 0.019	0.560 ± 0.012	0.0002	0.0014	0.8903	0.0010

* The global properties were calculated with one-way ANOVA test. If the results one-way ANOVA were significance, post-hoc analysis with least significant difference was subsequently applied.

Table S7. Nodal local efficiency compared between the patients and healthy groups

Node name	GRE group	non-GRE group	Health group	One-way ANOVA (p value)	GRE vs non-GRE (p value)	GRE vs Health (p value)	non-GRE vs n Health (p value)
A45c_L	0.331 ± 0.041	0.381 ± 0.050	0.348 ± 0.051	0.7486	-	-	-
A45c_R	0.371 ± 0.046	0.340 ± 0.052	0.340 ± 0.052	0.7904	-	-	-
A45r_L	0.373 ± 0.041	0.506 ± 0.051	0.376 ± 0.048	0.0783	-	-	-
A45r_R	0.312 ± 0.038	0.366 ± 0.052	0.347 ± 0.050	0.7096	-	-	-
A44op _L	0.365 ± 0.045	0.418 ± 0.052	0.269 ± 0.031	0.0567	-	-	-
A44op _R	0.307 ± 0.033	0.359 ± 0.040	0.303 ± 0.052	0.5839	-	-	-
A44v _L	0.344 ± 0.039	0.478 ± 0.045	0.328 ± 0.045	0.3179	-	-	-
A44v _R	0.285 ± 0.037	0.383 ± 0.053	0.347 ± 0.037	0.2717	-	-	-
A12/47l _L	0.336 ± 0.044	0.293 ± 0.045	0.322 ± 0.046	0.7864	-	-	-
A12/47l _R	0.364 ± 0.038	0.387 ± 0.048	0.370 ± 0.044	0.9277	-	-	-
A4hf _L	0.249 ± 0.032	0.334 ± 0.057	0.392 ± 0.045	0.0884	-	-	-
A41/42_L	0.437 ± 0.047	0.353 ± 0.040	0.307 ± 0.031	0.0696	-	-	-
A22c _L	0.370 ± 0.038	0.359 ± 0.049	0.333 ± 0.038	0.8174	-	-	-
A22r _L	0.302 ± 0.047	0.478 ± 0.053	0.321 ± 0.037	0.1554	-	-	-

A21c_L	0.325 ± 0.036	0.430 ± 0.042	0.389 ± 0.037	0.1531	-	-	-
aSTS_L	0.341 ± 0.044	0.384 ± 0.051	0.351 ± 0.043	0.7921	-	-	-
rpSTS_L	0.392 ± 0.043	0.416 ± 0.047	0.375 ± 0.042	0.8049	-	-	-
cpSTS_L	0.345 ± 0.054	0.381 ± 0.048	0.364 ± 0.045	0.8749	-	-	-
A40c_L	0.301 ± 0.032	0.455 ± 0.039	0.437 ± 0.037	0.0061	0.0102	0.0280	0.7239
A40c_R	0.350 ± 0.044	0.344 ± 0.043	0.400 ± 0.042	0.6034	-	-	-
A39rv_L	0.346 ± 0.041	0.464 ± 0.043	0.383 ± 0.046	0.1475	-	-	-
A39rv_R	0.428 ± 0.046	0.449 ± 0.052	0.426 ± 0.038	0.9242	-	-	-

* The global properties were calculated with one-way ANOVA test. If the results one-way ANOVA were significance, post-hoc analysis with least significant difference was subsequently applied.

Table S8. Degree centrality compared between the patients and healthy groups

Node name	GRE group	non-GRE group	Health group	One-way ANOVA (p value)	GRE vs non-GRE (p value)	GRE vs Health (p value)	non-GRE vs n Health (p value)
A45c_L	4.752 ± 0.331	4.375 ± 0.460	4.470 ± 0.308	0.7587	-	-	-
A45c_R	4.222 ± 0.350	3.687 ± 0.349	4.548 ± 0.328	0.2059	-	-	-
A45r_L	4.704 ± 0.388	4.518 ± 0.347	4.226 ± 0.347	0.6452	-	-	-
A45r_R	4.081 ± 0.190	3.538 ± 0.332	4.262 ± 0.301	0.1721	-	-	-
A44op _L	3.937 ± 0.284	4.355 ± 0.341	4.185 ± 0.369	0.6724	-	-	-
A44op _R	4.552 ± 0.378	4.117 ± 0.405	3.510 ± 0.312	0.1376	-	-	-
A44v _L	4.750 ± 0.250	5.821 ± 0.349	4.071 ± 0.262	0.0007	0.0282	0.6218	0.0005
A44v _R	3.885 ± 0.244	4.560 ± 0.433	4.339 ± 0.356	0.3910	-	-	-
A12/47l _L	4.498 ± 0.314	4.206 ± 0.450	4.046 ± 0.294	0.6672	-	-	-
A12/47l _R	4.228 ± 0.322	4.419 ± 0.321	4.121 ± 0.353	0.8146	-	-	-
A4hf _L	3.615 ± 0.295	3.244 ± 0.356	3.425 ± 0.283	0.7046	-	-	-
A41/42_L	4.262 ± 0.362	4.115 ± 0.316	4.776 ± 0.361	0.3721	-	-	-
A22c _L	4.304 ± 0.303	4.298 ± 0.336	4.123 ± 0.375	0.9129	-	-	-
A22r _L	4.190 ± 0.401	4.179 ± 0.404	4.556 ± 0.266	0.7063	-	-	-
A21c_L	4.510 ± 0.287	4.843 ± 0.390	4.526 ± 0.327	0.7339	-	-	-
aSTS_L	4.298 ± 0.306	4.464 ± 0.390	4.776 ± 0.363	0.6284	-	-	-
rpSTS_L	5.020 ± 0.317	4.952 ± 0.362	4.722 ± 0.279	0.7899	-	-	-
cpSTS_L	3.766 ± 0.349	4.728 ± 0.399	4.417 ± 0.347	0.1715	-	-	-
A40c_L	4.792 ± 0.340	5.014 ± 0.440	5.208 ± 0.311	0.7262	-	-	-

A40c_R	4.397 ± 0.400	4.397 ± 0.404	4.702 ± 0.333	0.8068	-	-	-
A39rv_L	5.012 ± 0.372	5.125 ± 0.338	4.540 ± 0.368	0.4776	-	-	-
A39rv_R	5.125 ± 0.339	3.948 ± 0.375	5.190 ± 0.292	0.1654	-	-	-

* The global properties were calculated with one-way ANOVA test. If the results one-way ANOVA were significance, post-hoc analysis with least significant difference was subsequently applied.

Table S9. Nodal clustering coefficient compared between the patients and healthy groups

Node name	GRE group	non-GRE group	Health group	One-way ANOVA (p value)	GRE vs non-GRE (p value)	GRE vs Health (p value)	non-GRE vs n Health (p value)
A45c_L	0.241 ± 0.031	0.290 ± 0.040	0.281 ± 0.043	0.6274	-	-	-
A45c_R	0.286 ± 0.040	0.284 ± 0.045	0.304 ± 0.033	0.9248	-	-	-
A45r_L	0.285 ± 0.032	0.412 ± 0.044	0.321 ± 0.046	0.0835	-	-	-
A45r_R	0.259 ± 0.032	0.316 ± 0.046	0.298 ± 0.044	0.6047	-	-	-
A44op _L	0.296 ± 0.039	0.323 ± 0.041	0.217 ± 0.025	0.0966	-	-	-
A44op _R	0.235 ± 0.025	0.283 ± 0.031	0.256 ± 0.049	0.6596	-	-	-
A44v _L	0.270 ± 0.032	0.355 ± 0.035	0.260 ± 0.039	0.1201	-	-	-
A44v _R	0.234 ± 0.029	0.301 ± 0.044	0.271 ± 0.029	0.3956	-	-	-
A12/47l _L	0.265 ± 0.037	0.223 ± 0.036	0.257 ± 0.038	0.6991	-	-	-
A12/47l _R	0.291 ± 0.032	0.316 ± 0.036	0.298 ± 0.038	0.8819	-	-	-
A4hf _L	0.201 ± 0.025	0.276 ± 0.047	0.329 ± 0.040	0.0641	-	-	-
A41/42_L	0.361 ± 0.044	0.286 ± 0.032	0.228 ± 0.021	0.2485	-	-	-
A22c _L	0.298 ± 0.032	0.286 ± 0.039	0.271 ± 0.033	0.8558	-	-	-
A22r _L	0.229 ± 0.037	0.386 ± 0.048	0.250 ± 0.027	0.0097	0.0150	0.7002	0.0433
A21c_L	0.255 ± 0.026	0.326 ± 0.034	0.296 ± 0.027	0.2389	-	-	-

aSTS_L	0.274 ± 0.036	0.307 ± 0.042	0.282 ± 0.040	0.8213	-	-	-
rpSTS_L	0.289 ± 0.034	0.326 ± 0.042	0.284 ± 0.038	0.7019	-	-	-
cpSTS_L	0.274 ± 0.047	0.291 ± 0.038	0.291 ± 0.037	0.9449	-	-	-
A40c_L	0.235 ± 0.025	0.348± 0.032	0.317 ± 0.031	0.2252	-	-	-
A40c_R	0.273 ± 0.036	0.282 ± 0.037	0.309 ± 0.037	0.7685	-	-	-
A39rv_L	0.250 ± 0.032	0.358 ± 0.035	0.312 ± 0.042	0.1168	-	-	-
A39rv_R	0.314 ± 0.035	0.371 ± 0.046	0.316 ± 0.029	0.4788	-	-	-

* The global properties were calculated with one-way ANOVA test. If the results one-way ANOVA were significance, post-hoc analysis with least significant difference was subsequently applied.

Reference

1. Calhoun VD, Wager TD, Krishnan A, et al. The impact of T1 versus EPI spatial normalization templates for fMRI data analyses. Hum Brain Mapp 2017;38:5331-5342
2. Ji GJ, Yu Y, Miao HH, et al. Decreased Network Efficiency in Benign Epilepsy with Centrotemporal Spikes. Radiology 2017;283:186-194
3. Humphries MD, Gurney K, Prescott TJ. The brainstem reticular formation is a small-world, not scale-free, network. Proc Biol Sci 2006;273:503-511
4. Gong Y, Wu H, Li J, et al. Multi-Granularity Whole-Brain Segmentation Based Functional Network Analysis Using Resting-State fMRI. Front Neurosci 2018;12:942

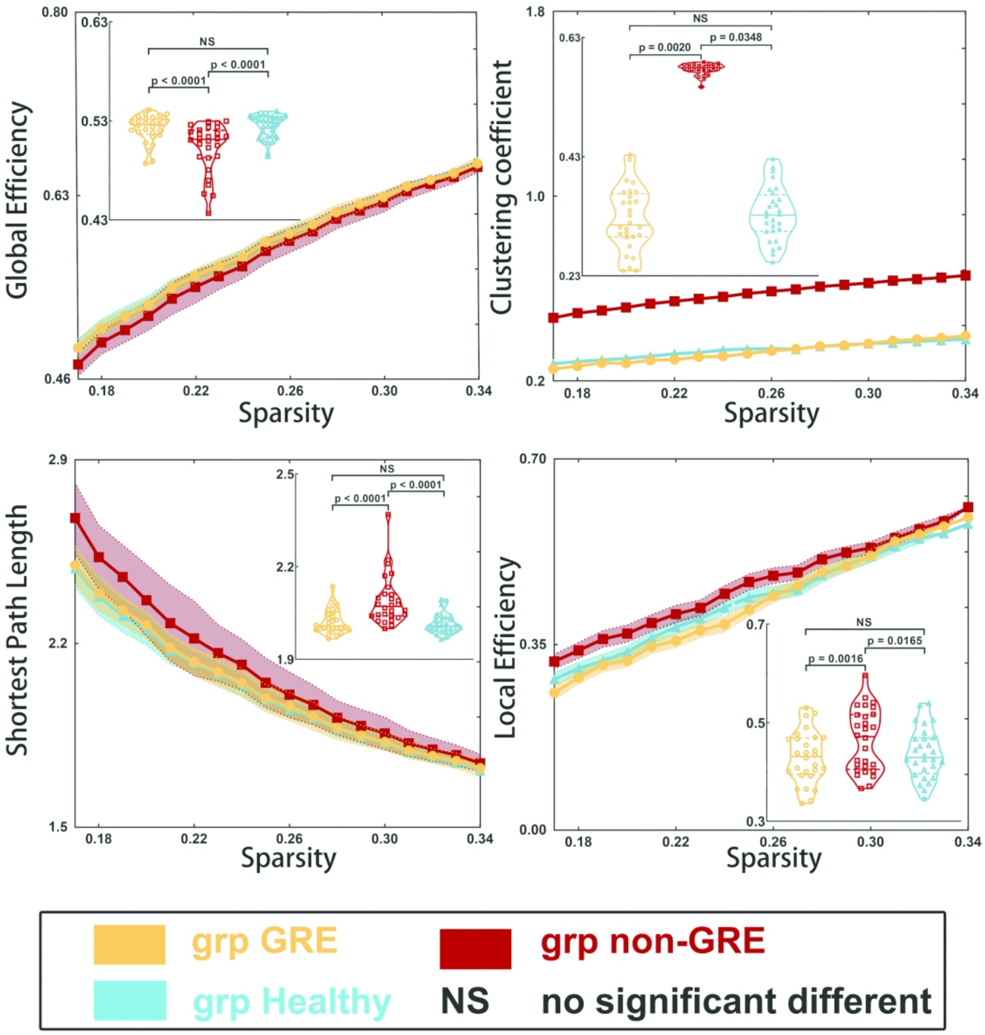

Figure 1. Results of alterations in global topological properties when gliomas grew in the right temporal lobe. The grp GRE = group of patients with glioma-related epilepsy. The grp non-GRE = group of patients without glioma-related epilepsy. The grp healthy = group of healthy participants.

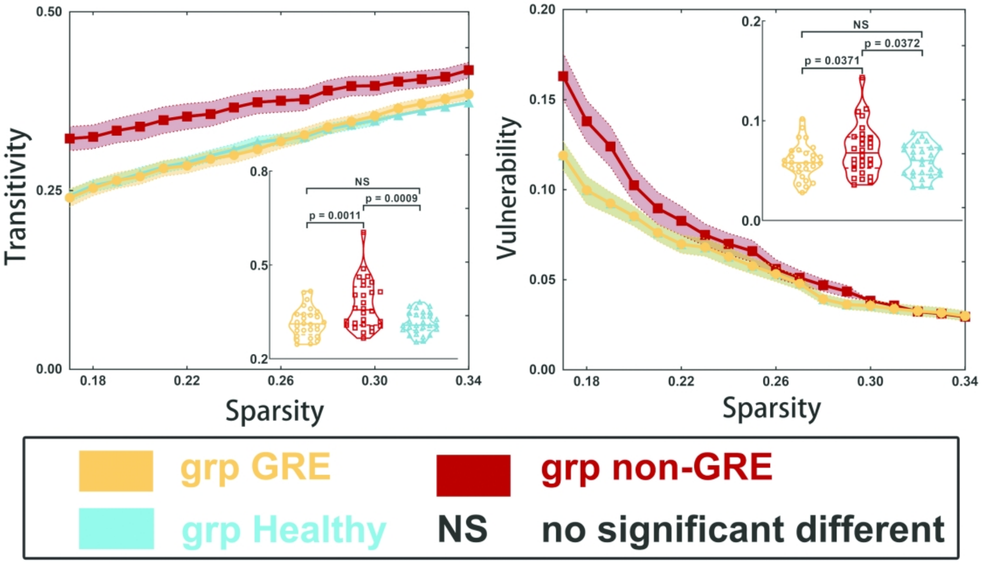

Figure 2. Results of alterations in transitivity and vulnerability when gliomas grew in the right temporal lobe. The grp GRE = group of patients with glioma-related epilepsy. The grp non-GRE = group of patients without glioma-related epilepsy. The grp healthy = group of healthy participants.

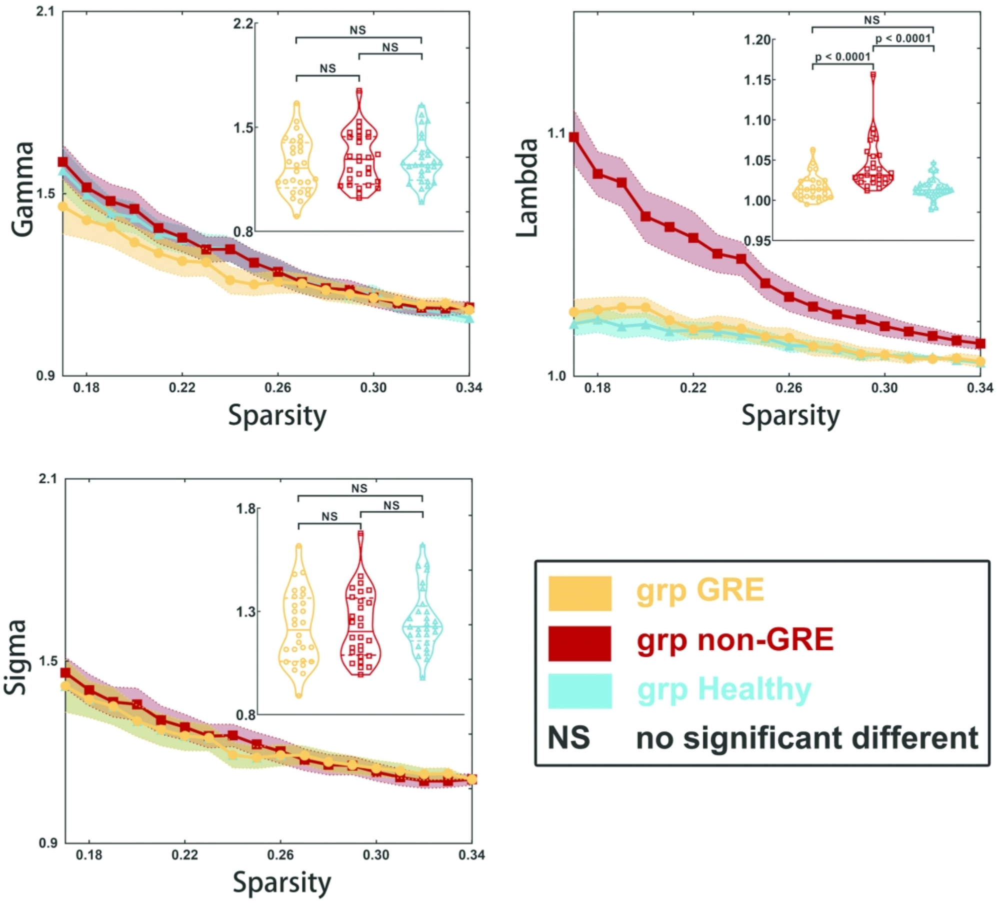

Figure 3. Results of alterations in small-worldness when gliomas grew in the right temporal lobe. The grp GRE = group of patients with glioma-related epilepsy. The grp non-GRE = group of patients without glioma-related epilepsy. The grp healthy = group of healthy participants.

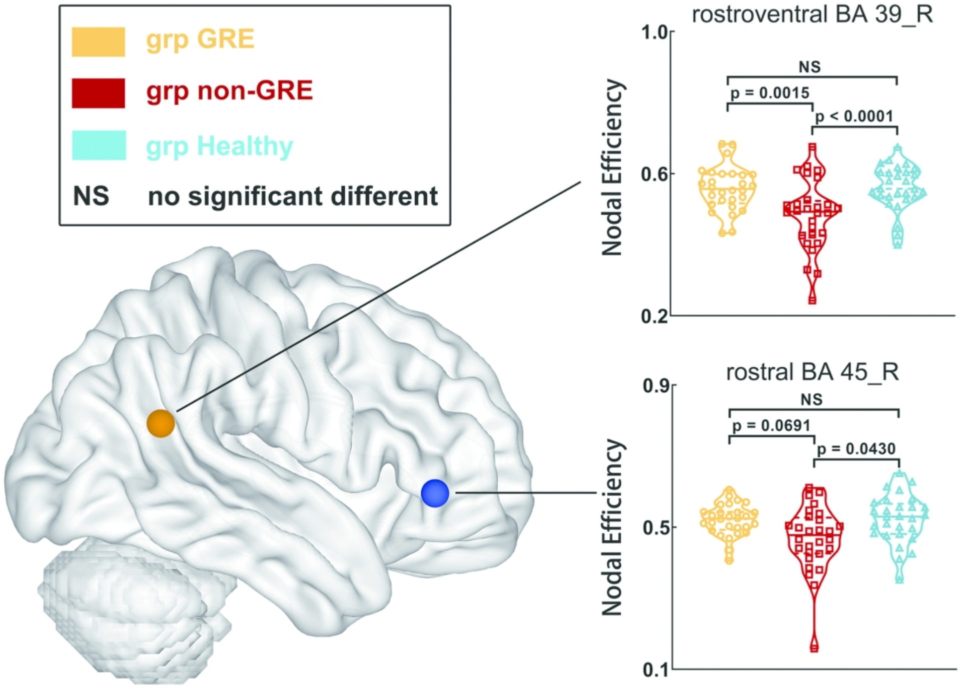

Figure 4. Results of alterations in nodal properties of right nodes in the language network when gliomas grew in the right temporal lobe. The grp GRE = group of patients with glioma-related epilepsy. The grp non-GRE = group of patients without glioma-related epilepsy. The grp healthy = group of healthy participants.

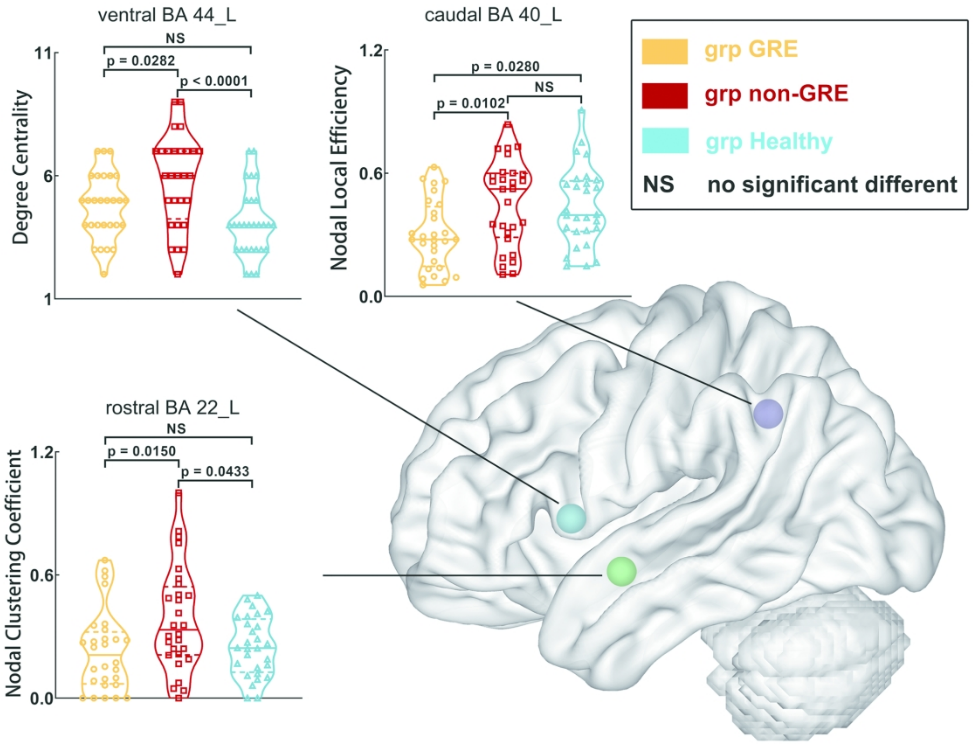

Figure 5. Results of alterations in nodal properties of left nodes in the language network when gliomas grew in the right temporal lobe. The grp GRE = group of patients with glioma-related epilepsy. The grp non-GRE = group of patients without glioma-related epilepsy. The grp healthy = group of healthy participants.
