# Supplementary material for: Epilepsy enhance global efficiency of language networks in right temporal lobe gliomas
Source: CNS Neurosci Ther. 2021 Jan 19;27(3):363–71. doi: 10.1111/cns.13595 (PMC7871790; doi:10.1111/cns.13595)
Supplement: Supplementary file 2 — App S1 [file CNS-27-363-s002.docx]

# Appendix S1

# Supplementary materials

# Part 1. Processing pipeline of rs-fMRI data.

The rs-fMRI data were processed as follows: a) transformation to a NIFTI file, b) removal of the first 5 time points, c) slice timing, d) realignment, e) normalization (normalized to EPI template [1]), f) smoothing (full width half maximum = 4 mm), g) temporal detrending (linear detrending), h) covariance regressing (white matter signal: with WMMask_3mm; CSF signal: with CSFMask_3mm; head motion: Friston—24 parameters), i) temporal filtering (0.01–0.08 Hz), and j) scrubbing (linear interpolation, subsequent time point number = 2, FD threshold = 0.5, and previous time point number = 1,).

# Part 2. Information of Topological Properties

# Clustering coefficient

Cluster coefficient represents the possibility that the neighbors of node *j* can interact with other nodes, and meant clustering degree of functional network. The formula was as follows:

$$Ci=\frac{2ej}{kj(kj-1)}$$

C*j*, cluster coefficient of node *j*; k*j*, the number of probable edges connecting to other nodes; e*j*, the number of actual edges connecting to other nodes.

# Global efficiency

Global efficiency meant the ability of information transmission at global level. The formula was as follows: (reference to supplementary materials of Ji G et al, Radiology, 2017 [2]):


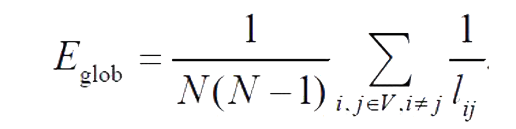


E*glob*, global efficiency of whole functional network; N, the number of actual edges connecting node *i* to other nodes in the whole network; l*i, k*, the shortest path length between the nodes j and k.

# Shortest path length

The shortest path length meant the minimum number of passing edges for information conduction between each two nodes. It describes the optimal pathway for information transmission at global level. The calculation formula was as follows:


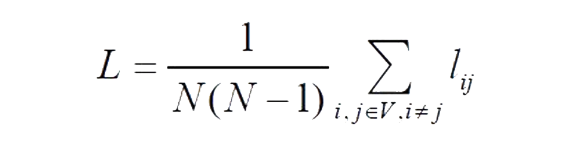


L, shortest path length of network; N, the number of actual edges connecting the node *i* to another node in the whole network; l*i, k*, the shortest path length between the node j and node k.

# Local efficiency

Local efficiency represents the ability of information conduction in local network. The calculation formula as follows:


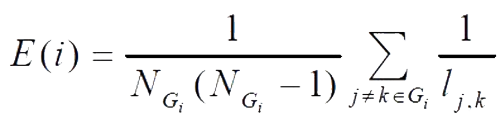


E*i*, local efficiency of local network; NG*i*, the number of actual edges connecting node *i* to other nodes in the local network; l*i, k*, the shortest path length between the nodes j and k.

# Nodal efficiency


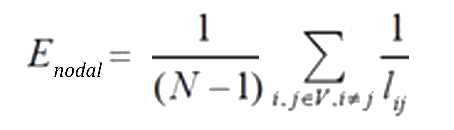
 Nodal efficiency represents the ability of information transmission of the node i. The calculation formula as follows:

E*_nodal_*, nodal efficiency of whole functional network; N, the number of actual edges connecting node *i* to other nodes in the whole functional network; l*i, k*, the shortest path length between the nodes j and k.

# Small-worldness properties

Small-worldness properties (gamma, lambda, and sigma) mean the efficiency of information transmission. Gamma (γ) = C_real_/C_random_ >> 1 (C = cluster coefficient). Lambda (λ) = L_real_/L_random_ ~ 1 (L = shortest path length). And Sigma (σ) = γ/λ > 1. [3, 4] If a network has a high value of sigma, the ability of information transmission will be strong.
